# Novel aminothiazole grafted cellulose composite for efficient removal of Hg(II) from wastewater in single and multicomponent systems

**DOI:** 10.1038/s41598-025-31905-2

**Published:** 2025-12-29

**Authors:** Magda A. Akl, Aya G. Mostafa

**Affiliations:** Department of Chemistry, Faculty of Science, Mansoura University, Mansoura, 31556 Egypt

**Keywords:** Hg(II), Heavy metals, Schiff base, Multicomponent study, Plackett–Burman design (PBD), ANOVA, Cellulose, Water treatment, Chemistry, Environmental sciences, Materials science

## Abstract

The pollution of mercury is a widespread and growing problem, as it poses significant environmental and health issues. Investigating adsorbents with high adsorption capacity is an urgent need to solve this issue. The current investigation introduces a novel aminothiazole Schiff base grafted cellulose (DAC@AHTT) adsorbent for Hg(II) removal from various water samples. The DAC@AHTT adsorbent was synthesized by the modification of dialdehyde cellulose (DAC) with 4-amino-5-hydrazinyl-4 H-1,2,4-triazole-3-thiol (AHTT) Schiff base. Moreover, the prepared DAC@AHTT adsorbent was characterized by physical, chemical, and morphological techniques, and its performance was systematically obtained. The maximum adsorption capacity of 495.7 mg.g^− 1^ was achieved at the optimum conditions of pH (6–8), a dose of 0.002 g, and 240 min. Hg(II) adsorption follows pseudo-2nd -order kinetics and Langmuir isotherm models, revealing the spontaneous, exothermic, and monolayer chemisorption. The DAC@AHTT material was successively employed for the Hg(II) removal from real water samples, with a recovery (Re, %) exceeding 98%. The DAC@AHTT adsorbent has a good recycling efficiency of 85% after three adsorption-desorption cycles. The adsorption mechanism of Hg(II) onto DAC@AHTT adsorbent has been elucidated, showing that N and S atoms are the main binding sites. The adsorbent was successfully employed for the adsorption of Hg(II), Ni(II), Pb(II), and Cu(II) in multicomponent systems by applying the Plackett-Burman design of experiments (PBD). These findings suggest that DAC@AHTT is a promising, efficient, and sustainable adsorbent for water treatment applications.

**Supplementary Information:**

The online version contains supplementary material available at 10.1038/s41598-025-31905-2.

## Introduction

 The rapid growth of industry, population, and civilization leads to significant water contamination^1^. Water contamination resulting from industrial effluents is a significant environmental concern. Discharging untreated industrial wastewater into various water bodies makes them unsuitable and unhealthy for irrigation, drinking, marine life, and domestic use^2–4^.

Mercury (Hg) is a naturally occurring silver-white liquid metal that has three oxidation states, 0, 1^+^, and 2^+^^5–7^. Mercury gains scientists’ attention among other environmental contaminants, as it is utilized in several industries. These include mining, medical uses, painting (as vermilion (HgS)), electrical devices, batteries, etc^8–11^. Despite its uses, mercury poses many toxic effects on the environment and living organisms. Its environmental persistence and bioaccumulation within living organisms can cause such dangerous illnesses. It disrupts cellular processes^12,13^. Several bacterial classes, including methanogens, can convert inorganic mercury into a more toxic organic form, leading to severe central nervous system harm^14^. Due to the risk of chronic poisoning, the World Health Organization (WHO) and the U.S. Environmental Protection Agency (EPA) have recommended maximum acceptable concentrations of Hg(II) in drinking water to be 0.002 mg.L^− 1^ and 0.001 mg.L^− 1^, respectively^15,16^.

Several techniques have been employed to address the Hg(II) water contamination issue^14^. These techniques include photocatalytic degradation^17^, bioremediation^18^, adsorption^14^, electrochemical techniques^19^, membrane filtration^20^, and ion exchange^21^. The advantages and disadvantages of some of these techniques are presented in Table 1^22–24^. Adsorption is considered a more desirable technique for removing heavy metals among the other methods mentioned. It has ease of use, high efficiency, affordability, and the capacity to manage large-scale systems^25–27^. Therefore, a crucial challenge in the adsorption process is the development of effective and readily available materials to be employed as adsorbents^28^. Numerous materials have been employed as effective adsorbents, including biochar^29^, cellulosic materials^30^, clay^31^, nanometal oxides^32^, activated carbon^33^, silica^34^, and metal-organic frameworks^35^.

Despite the adsorption advantages, there are some limitations, including the presence of multiple ions in real wastewater, which results in a decrease in removal efficiency. Moreover, the reuse of adsorbents may be another challenge. These drawbacks underscore the need to develop resilient, selective, and readily regenerable adsorbents that can maintain high performance in challenging real-world scenarios^36^.

Cellulose is a naturally occurring polymer that can be derived from many natural sources, like cotton, bamboo, flax, and straw. It has numerous advantages due to its reusability, affordability, availability, and eco-friendliness^37–39^. However, its lack of functional groups leads to a relatively low pollutant adsorption efficiency. To increase its ability to adsorb various contaminants with high adsorption efficiency, chemical modification is introduced through the addition of new, desired functional groups^14^. Cellulose has a high ability to be modified through esterification^40^, oxidation^41^, etherification^42^, or grafting^43^ as it has an abundance of OH groups. Moreover, cellulose modification enhances its ability to remove various contaminants^44^. Cellulose modification utilizing a thio group-containing ligand increases the adsorption efficiency^45,46^.

Several published investigations have utilized cellulose-based adsorbents to eliminate different pollutants. These pollutants include Hg(II)^14^, As^47^, uranium (VI)^48^, Cr(IV)^49^, Cu(II)^50^, and Hg(II), Cd(II), Cu(II), and Pb(II)^51^. The oxidized microfibers, dialdehyde cellulose, have been applied to remove Eriochrome black T dye^52^. The Cu(II), Hg(II), and Pb(II) were removed using guanylthiosemicarbazide-functionalized oxidized cellulose^53^. Moreover, the functionalized Flax fibers with the semicarbazide were utilized to eliminate both Cr(VI) and Alizarin Red S dye from various water samples^54^. For selective Hg(II) removal, Mostafa et al. modified the DAC by utilizing cyanoacetohydrazide and CS_2_, respectively^14^.

The AHTT ligand has been utilized in the current investigation as it has a functionalized structure. It is rich in amino groups of the 1,2,4-triazole ring and hydrazine, as well as the thiol group. This molecular structure offers efficient moieties that support the cellulose surface, featuring multiple active sites for effective heavy metal adsorption^55^. They are considered to be affordable, simple to prepare, safe, and ecologically beneficial compounds^56–58^. Molecules that have a 1,2,4-triazole moiety are strong N-atom donors towards d-metal ions and can be easily deprotonated^59^.

Recently, various studies have developed ligand-based adsorbents for removing pollutants from water^60–62^. Many investigations have synthesized ligand-modified mesoporous silica for use as adsorbents for removing various water pollutants, including Yb(III), Cd(II), Pb(II), Ni(II), Ce(III), and Sm(II)^63–67^. These adsorbents achieved good adsorption capacities. However, despite their performance, many of these investigations had some limitations, such as the high cost of materials, challenges in large-scale applications, and difficulty in obtaining them, as some are not locally available. Moreover, the synthesis of some of them requires highly optimized conditions, which could make them less scalable and practical. In contrast, the ligand used in our investigation is considered a cost-effective, easy-to-prepare, safe, and ecologically beneficial compound. It has antioxidant, antibacterial, antianxiety, antitubercular, anti-inflammatory, and anti-cancer properties^56–58^. This study contributes to the development of an efficient and affordable adsorbent that can be applied on a large scale.

The current investigation’s novelty lies in the synthesis of a cellulose-based material (DAC@AHTT) rich with sulfur and amine groups through the DAC modification with the AHTT Schiff base. This dual-functionalization technique enhances the adsorption affinity for Hg(II) and other heavy metals. Moreover, this is the first study to apply DAC@AHTT for the simultaneous adsorption of Hg(II), Pb(II), Cu(II), and Ni(II) from multicomponent systems and the removal of Hg(II) from single-metal systems. Furthermore, the Plackett–Burman Design (PBD) was employed to systematically optimize and assess the multicomponent adsorption process, providing a statistically sound understanding of the variables influencing adsorption efficiency. An innovative approach to the development of effective and sustainable materials for heavy metal remediation is this integrated experimental–statistical approach.

This investigation has many advantages. The current cellulose modification method is straightforward, cost-effective, and suitable for large-scale production. The DAC@AHTT adsorbent exhibits a high adsorption capacity for Hg(II) from various real water samples, demonstrates a good ability to remove heavy metals in multicomponent systems, and displays satisfactory recycling performance over three consecutive adsorption-desorption cycles.

Consequently, the subsequent goals were used to assess the performance and value of the present investigation: (i) Preparation and characterization of the novel DAC@AHTT adsorbent utilizing FTIR, BET, SEM, CHNS, and EDX techniques; (ii) Studying the optimum parameters of Hg(II) adsorption to achieve optimum Hg(II) adsorption capacity, including solution pH, ionic strength, and temperature, the initial Hg(II) concentration, the DAC@AHTT mass, and the oscillation period; (iii) Investigation of the isotherm, kinetics, statistical error functions, and thermodynamic parameters for the Hg(II) adsorption onto DAC@AHTT adsorbent; (iv) The use of DAC@AHTT adsorbent for efficient removal of Hg(II) from diverse polluted samples; (v) Comparison of the efficiency of DAC@AHTT adsorbent toward Hg(II) with other previously documented adsorbents; (vi) Studying the reusability of DAC@AHTT adsorbent to investigate the stability of the structure of DAC@AHTT adsorbent; (vii) Elucidation of the Hg(II) adsorption mechanism onto DAC@AHTT adsorbent; VIII. Evaluation of the combined effect of Hg(II), Ni(II), Pb(II), and Cu(II) on each other removal by the DAC@AHTT composite; ix. Operating statistical tools such as Plackett-Burman design (PBD) of experiment and ANOVA to optimize the process parameters to determine the antagonistic effect of Hg(II), Ni(II), Pb(II), and Cu(II) on each other’s removal in the multi contaminated system and to interpret the significance and effect of these metals on each other’s removal, respectively.

**Table 1:** Advantages and disadvantages of Hg(II) removal techniques.

Technique	Advantages	Disadvantages
Membrane filtration	Controlled selectivity, as it depends on the type of membrane. Simple and non-toxic materials.	Not specific for Hg High cost Low selectivity Fouling Instability under high pressure and high Hg(II) concentrations
Photocatalytic degradation	Inexpensive (depending on the applied catalyst).	The volatile Hg(0) formation (toxic and needs trapping)
Ion exchange	Tends to be low cost (when utilizing natural zeolites). Efficient (when utilizing thio-based resins).	Requires a pretreatment step High cost of resins Resins used during the process require chemical regeneration, producing secondary pollution.
Phytoremediation bio- remediation	Less harmful by-products Cheap	For live microorganisms, it is ineffective when the metal concentration is high. May has effects on plant growth and photosynthesis ability Sensitive to the operational environment
Electrolytic	High selective	High costs Consume large amounts of energy
Adsorption	Cost effective Easy to operate Highly efficient High adsorption rates Availability of a wide selection of adsorbents	Low selectivity

## Experimental

### Materials

Cellulose powder, mercury chloride (HgCl_2_, 99%), nickel acetate (Ni(CH₃CO₂)₂0.4 H₂O, 98%), lead acetate (Pb(NO_3_)_2_, ≥ 99.0%), copper chloride (CuCl_2_.2H_2_O, ≥ 99.0%), HCl (37%), NaOH (99.99%), H_2_SO_4_ (95.0–98.0%), ethanol (≥ 99.5%), methanol (≥ 99.8%), and NaCl (*≥* 99*.5*%) were bought from Sigma Aldrich. The ligand 4-Amino-5-hydrazinyl-4 H-1,2,4-triazole-3-thiol hydrochloride 95% (AHTT, C_2_H_7_ClN_6_S) was purchased from BLD Pharm Company, China.

### Characterization

The pH_PZC_ of DAC@AHTT adsorbent was investigated as follows: 0.1 g of the DAC@AHTT adsorbent was added to 25 mL NaCl (0.01 mol.L^− 1^) solutions at a pH range of (2–12), which was adjusted using HCl (0.1 mol.L^− 1^) and NaOH (0.1 mol.L^− 1^). The prepared mixtures were shaken for 48 h in the equilibrated shaker. Then, the final pH (pH_f_) was measured, and ΔpH was calculated as in Eq. (1). ΔpH, y-axis, was plotted against the initial pH (pH_i_), x-axis, to calculate the pH_PZC_ value (ΔpH = 0)^30^.

1 $$\Delta pH={pH}_{i}-{pH}_{f}$$

Fourier transform infrared, FT-IR, spectra of DAC, DAC@AHTT adsorbent, DAC@AHTT-Hg(II), and the DAC@AHTT-Cu(II) samples were estimated utilizing a Perkin Elmer at 4000 –400 cm^− 1^ wavenumber range using potassium bromide pellets. The surface morphologies of the DAC, DAC@AHTT, and DAC@AHTT-Hg(II) were examined by scanning electron microscopy (A JSM-6510LV), which was also utilized to evaluate the EDX (Energy dispersive X-ray spectroscopy) spectral investigation of the DAC@AHTT-Hg(II) sample. The specific surface area of S_BET_, and pore diameter of native cellulose and DAC@AHTT adsorbent were estimated using the Brunauer Emmet Teller, BET, analysis (Size Analyzer QUANTACHROME - NOVA 2000 Series). An elemental analyzer, Costech ECS-4010, investigated the CHNS composition of native cellulose and DAC@AHTT adsorbent. The residual investigated heavy metals’ concentration was investigated using ICP-OES (Agilent’s 5100 equipment) at the optimal parameters mentioned in Table S1.

### The material safety data sheets (MSDS)

The Material Safety Data Sheets (MSDS) and supplier-provided safety documentation were used to assess each chemicals used in this investigation. A summary table classifying the risks, safety measures, and appropriate handling techniques for each substance is presented in Table S2. Strict adherence to appropriate laboratory procedures was maintained, including the use of personal protective equipment (PPE), ensuring adequate ventilation, and following safe storage and disposal protocols. There was no intentional release of dangerous materials during the application or synthesis procedures.

### Preparation of the DAC@AHTT adsorbent

**Fig. 1:**
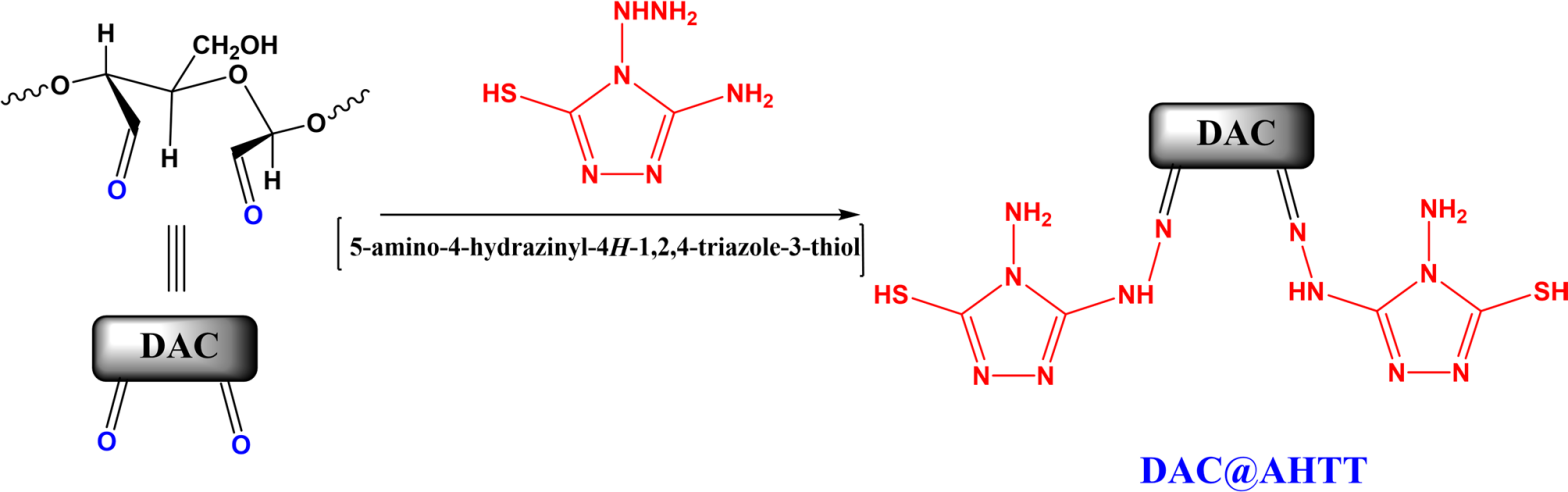
Preparation of DAC@AHTT adsorbent.

Initially, dialdehyde cellulose (DAC) with an aldehyde content of 38.4% was prepared as reported in the literature^14^. One gram of DAC was added to a methanolic solution of AHTT with one drop of H_2_SO_4_. Then, this mixture was refluxed for 4 h at a temperature of 70 °C, followed by stirring overnight at room temperature to form the DAC@AHTT adsorbent. Then, it was filtered and washed with methanol, followed by ethanol to remove any excess of unreacted AHTT. Finally, the obtained DAC@AHTT adsorbent was dried at 45^ο^C. The preparation of DAC@AHTT adsorbent is illustrated in Fig. 1.

**Fig. 2:**
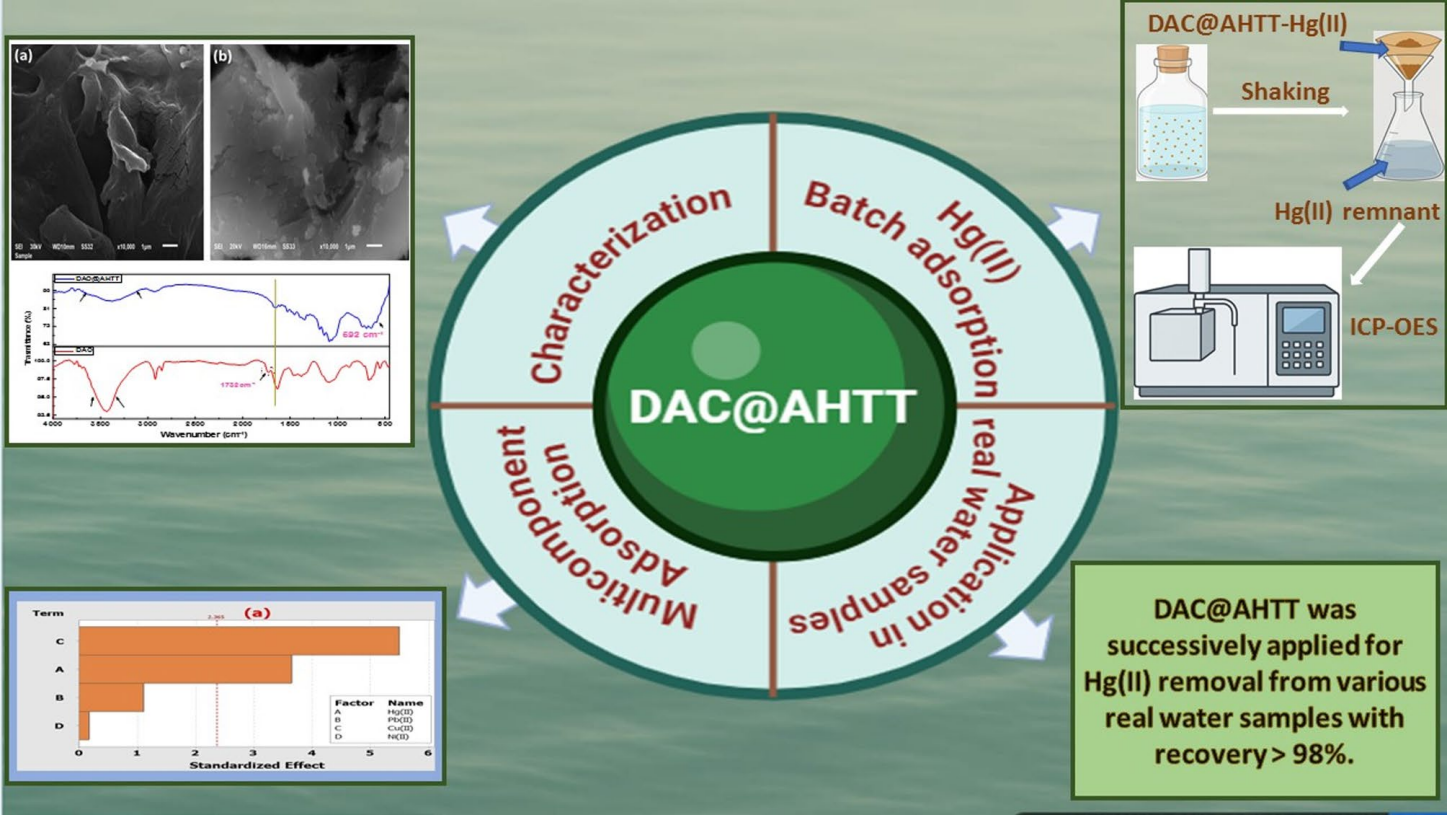
Synthesis, characterization of DAC@AHTT, and its use for the removal of Hg(II).

The synthesis, characterization of DAC@AHTT adsorbent, and its use for the removal of Hg(II) are graphically represented in Fig. 2.

### Batch adsorption

#### Removal of Hg(II) in a single-component study

The Hg(II) adsorption using the DAC@AHTT adsorbent was studied through batch adsorption experiments in 125 mL stoppered bottles containing 10 mL of Hg(II) solution and a specified amount of DAC@AHTT adsorbent. Various parameters were investigated, including pH (ranging from 2 to 10), metal ion concentration (25–400 mg.L^-1^), DAC@AHTT adsorbent weight (0.001–0.02 g), and oscillation time (15–300 min). Unless otherwise noted, the pH, DAC@AHTT adsorbent dose, Hg(II) concentration, and oscillating time were fixed to 6, 0.002 g, 100 mg.L^-1^, and 240 min, respectively. Diluted solutions of HCl and NaOH were used to adjust the pH of the Hg(II) solutions. Further, the samples were collected, filtered, and analyzed for residual Hg(II) concentrations using ICP-OES (Agilent’s 5100 equipment).

The Hg(II) removal (R, %) and adsorption efficiency at equilibrium (q_e_, mg.g^-1^) were calculated by applying Eqs. (2) and (3), respectively^31^.

2 $$\mathrm{R\%}=\frac{{\mathrm{C}}_{\mathrm{i}}-{\mathrm{C}}_{\mathrm{f}}}{{\mathrm{C}}_{\mathrm{i}}}\times 100$$

3 $${\mathrm{q}}_{\mathrm{e}}=\frac{\left({\mathrm{C}}_{\mathrm{i}}-{\mathrm{C}}_{\mathrm{f}}\right)*\mathrm{v}}{m}$$

Where C_i_ (mg.L^− 1^) and C_f_ (mg.L^− 1^) are the initial and final concentrations of the Hg(II). m (g) and V(L) are the DAC@AHTT adsorbent dose and the Hg(II) solution volume.

#### Removal of Hg(II) from the multi-contaminated system

For examining the removal of heavy metals in a multi-contaminated system, a Plackett-Burman experimental design (PBD) with 12 experimental runs, featuring various combination levels of Hg(II), Ni(II), Pb(II), and Cu(II), was selected (Table 2). The lower and upper limits of the concentration for each of Hg(II), Ni(II), Pb(II), and Cu(II) were selected as 25 and 50 mg.L^− 1^.

The Plackett–Burman experimental approach was applied to assess the comparative significance of four factors^68,69^. Established on the Plackett–Burman factorial design, each factor was analyzed at two levels: ʻ-1ʼ for the low level (25 mg.L^-1^) and ʻ+1ʼ for the high level (50 mg.L^-1^). Moreover, the matrix design of the tested factors was vetted in 12 experimental trials. All trials were conducted using DAC@AHTT adsorbent to investigate the effect of various variables on the adsorption process, either positively or negatively (inhibitory). The four studied variables were Hg(II), Pb(II), Cu(II), and Ni(II). At room temperature, 0.01 g of DAC@AHTT adsorbent was mixed with the investigated mixed metals solution for 4 h at a pH of 6. Plackett–Burman design is established on a first-order model as presented in Eq. (4)^70^.

4 $$Y={\beta }_{0}+\sum {\beta }_{i}{X}_{i}$$

Where, Y is the expected response, is the DAC@AHTT adsorption capacity (mg.g^-1^). $\:{\beta\:}_{i}$ is the exhibited coefficient of the coded level, X_i_.

**Table 2:** Plackett–Burman experimental design matrix showing the various combination levels of the heavy metals in the multicomponent removal study by DAC@AHTT.

Trials	Investigated metals coded values			
	Hg(II)	Pb(II)	Cu(II)	Ni(II)
1	+ 1	-1	+ 1	-1
2	+ 1	+ 1	-1	+ 1
3	+ 1	+ 1	+ 1	+ 1
4	+ 1	-1	+ 1	+ 1
5	+ 1	+ 1	-1	+ 1
6	+ 1	+ 1	+ 1	-1
7	-1	+ 1	+ 1	+ 1
8	-1	-1	+ 1	+ 1
9	-1	-1	-1	+ 1
10	+ 1	-1	-1	-1
11	-1	+ 1	-1	-1
12	-1	-1	-1	-1

### Adsorption isotherms, kinetic, statistical error validity, and thermodynamic investigations

#### Adsorption isotherms

The adsorption isotherm can be described as follows: the relation between the pollutant adsorbed amount (mg.g^− 1^) on the studied adsorbent surface and the unadsorbed, equilibrium concentration (mg.L^− 1^), at a constant temperature. The maximum adsorption efficiency (q_e_, mg.g^− 1^) of Hg(II) and binding affinity can be estimated by applying the Langmuir and Freundlich models in their linear form. The obtained parameters (q_m_, K_F_, K_L_, and n) were estimated as shown in Eqs. (5) and (6), respectively. The Langmuir and Freundlich isotherms, in their nonlinear forms, are represented by Eqs. (7) and (8), respectively.

5 $$\frac{{\mathrm{C}}_{\mathrm{e}}}{{\mathrm{q}}_{\mathrm{e}}}=\frac{1}{{\mathrm{k}}_{\mathrm{L}1}{\mathrm{q}}_{\mathrm{m}}}+\frac{{\mathrm{C}}_{\mathrm{e}}}{{\mathrm{q}}_{\mathrm{m}}}$$

6 $$\mathrm{ln}{q}_{e}=ln{K}_{f1}+\frac{1}{n}ln{C}_{e}$$

7 $${q}_{e}=\frac{{q}_{m}{K}_{L2}{C}_{e}}{1+{K}_{L2}{C}_{e}}$$

8 $${q}_{e}={K}_{f2}{C}_{e}^{1/n}$$

C_e_ (mg.L^− 1^) and 1/n are the Hg(II) equilibrium concentration and the heterogeneity factor, respectively. K_L_ (L.mg^− 1^) and K_F_ ((mg.g^− 1^)(L.mg^− 1^)^1/n^) are Langmuir and Freundlich constants, respectively. Both q_e_ and q_m_ are expressed in mg.g^− 1^ and defined as the Hg(II) capacity at equilibrium and the adsorption maximum uptake.

#### Adsorption kinetics

Pseudo-1st -order (PFO), pseudo-2nd -order (PSO), Intraparticle diffusion (IPD), and Boyd kinetic models were studied to evaluate the Hg(II) adsorption rate-limiting step, as shown in Eqs. (9–12), respectively.

9 $$1/{{\mathrm{q}}_{\mathrm{t}}}_{(\mathrm{ads})}= {\mathrm{k}}_{1}/{{\mathrm{q}}_{\mathrm{e}}}_{(\mathrm{ads})}\text{t }+ 1/{{\mathrm{q}}_{\mathrm{e}}}_{(\mathrm{ads})}$$

10 $$\mathrm{t}/{\mathrm{q}}_{\mathrm{t}}(\mathrm{ads})=1/{\mathrm{k}}_{2}{{\mathrm{q}}_{\mathrm{e}}^{2}}_{(\mathrm{ads})}+ (1/{{\mathrm{q}}_{\mathrm{e}}}_{(\mathrm{ads})})\mathrm{t}$$

11 $$q_{t} = k_{{{\mathrm{int}} }} t^{0.5} + c$$

12 $$F\left( t \right) = 1 - \frac{6}{{\pi^{2} }}\mathop \sum \limits_{n = 1 }^{\infty } \frac{1}{{ n^{2} }} \exp \left( { - n^{2} Bt} \right)$$

The Hg(II) adsorption efficiency at equilibrium and at a particular time t (min) are represented as q_e_ (mg.g^− 1^) and q_t_ (mg.g^− 1^), respectively. As well as K_1_ and K_2,_ there are PFO and PSO, respectively. K_int_ was the rate constant for IPD (mg.(g.min^1/2^)^−1^ and C represents the boundary layer thickness, $\:F$ is the fraction of equilibrium at different times (t), and $\:B\:\left(t\right)$ is the mathematical function of$\:\:F$. *n* is an integer that defines the infinite series solution, and $\:F\:$is the equilibrium fractional attainment at time $\:t$.

#### Statistical error validity on isotherm and kinetic models (The goodness of fit)

Various error functions were employed to investigate and evaluate the well-fitted isotherm and kinetic models, aiming to minimize the error dispersion between the calculated values obtained from theoretical model correlations and experimental values. The goodness of fit was estimated employing the following error functions: The chi-square statistic (χ^2^), the sum of squares error (SSE), and the mean square error (MSE), which are shown in Eqs. (13–16), respectively^31^.

13 $$\chi^{2} = \mathop \sum \limits_{i = 1}^{n} \frac{{\left( {q_{e i} \exp - q_{e i} cal} \right)^{2} }}{{q_{e i} cal}}$$

14 $$SSE = \mathop \sum \limits_{i = 1}^{n} \left( {q_{e i} \exp - q_{e i} cal} \right)^{2}$$

15 $$MSE = \frac{1}{{N_{\exp } }}\mathop \sum \limits_{i = 1}^{n} \left( {q_{e i} \exp - q_{e i} cal} \right)^{2}$$

16 $$HYBRID = \frac{100}{{N_{\exp } - N_{parameter} }}\mathop \sum \limits_{i = 1}^{n} \frac{{q_{e i} \exp - q_{e i} cal}}{{q_{e i} \exp }}$$

The symbol n denotes the number of incorporated observations. The subscripts *“cal”* and *“exp”* refer to theoretically calculated and experimental data, respectively.

#### Thermodynamicstudies

To investigate the nature of the Hg(II) adsorption process onto the DAC@AHTT surface in terms of spontaneity and feasibility and to assess the degree of randomness at the solid ‘’adsorbent’’ and liquid interface, adsorption thermodynamic parameters (ΔG°ads, ΔH°ads, and ΔS°ads) were determined at a temperature range of 298–318 K. Where ΔG°ads, ΔH°ads, and (ΔS°ads) are the Free energy, the heat of enthalpy, and adsorption entropy, respectively. Hg(II) adsorption by DAC@AHTT material was determined, and the ΔG°ads parameter was calculated from the following equations: Eqs. (17–20). The plotting of lnK_e_ vs 1/T, the temperature in Kelvin, for Hg(II) adsorption onto the DAC@AHTT material is demonstrated in Fig. 11.

17 $${\mathrm{K}}_{\mathrm{C}}= {\mathrm{C}}_{\mathrm{ad}}/{\mathrm{C}}_{\mathrm{e}}$$

18 $${K}_{e}={K}_{c}\times Adsorbate Mwt$$

19 $$\Delta G_{adsn}^{o} = - RT \ln K_{C}$$

20 $$\ln K_{e} = \Delta S^{o}_{adsn} /R - \Delta H_{adsn}^{o} /RT$$

As K_C_, R, C_ad_, and C_e_ are thermodynamic equilibrium constants (L.g^− 1^), universal gas constant, the Hg(II) concentration taken by DAC@AHTT at equilibrium (mg.g^− 1^), and the Hg(II) concentration at equilibrium (mg.L^− 1^), respectively. K_e_ is a dimensionless thermodynamic constant^71^.

### Desorption and regeneration

The reusing of DAC@AHTT adsorbent after the Hg(II) loading on its surface was acquired through three subsequent cycles of adsorption/desorption. The adsorption experiment was obtained utilizing 0.002 g of the DAC@AHTT and 10 mL solution of 100 mg.L^− 1^ Hg(II) at a pH of 6 for 4 h. For the desorption experiment, 10 mL of eluent solution, composed of 0.1 mol.L^− 1^ HNO_3_ and 0.1 mol.L^− 1^ thiourea (1:1), was mixed with 0.01 g of DAC@AHTT-Hg(II) and then shaken for 4 h^14^. Ultimately, three additional adsorption-desorption cycles were performed using the regenerated DAC@AHTT. The Hg(II) desorption (D, %) from the DAC@AHTT adsorbent was calculated as presented in Eq. (21).

21 $$\text{D }(\mathrm{\%})= \frac{\text{ Hg}\left(\mathrm{II}\right)\text{desorbed amount to the solution }(mg.{g}^{-1})}{\text{amount of Hg}(\mathrm{II})\text{ adsorbed }(mg.{g}^{-1})}\times 100$$

### Applications

Four different water samples (tap, waste, Nile, and sea) were utilized to investigate and evaluate the applicability of the prepared material (DAC@AHTT). The organic matter in the investigated water samples was digested as follows: a mixture composed of 0.5 g K_2_S_2_O_8_ and 5 mL of H_2_SO_4_ (95.0–98.0% (w/w)) was added to each water sample, followed by heating at 90 °C for 2 h. Various concentrations, ranging from 5 to 100 mg.L^-1^, of Hg(II) were spiked into the digested samples. Then, spiked samples were mixed with 0.002 g of DAC@AHTT, and the pH value was adjusted to 6 with continuous shaking for 4 h. After that, the solutions were centrifuged, and an additional 0.002 g of DAC@AHTT was added to the supernatant to ensure the complete separation of analytes. Finally, the remaining Hg(II) was assessed by utilizing ICP-OES.

## Results and discussion

### Physicochemical properties of DAC@AHTT adsorbent

The adsorbent’s specific surface area, S_BET_, is a crucial factor in the adsorption process, as it significantly affects the adsorbent’s capacity for metal ions. The surface areas of DAC and DAC@AHTT adsorbents were measured and are shown in Table 3. The modification increased the surface area by the AHTT ligand that contains thiol and amine groups to 42.4 m^2^.g^− 1^. This increase may be due to the incorporation of the bulky AHTT ligand into cellulose. The pore diameter of DAC@AHTT is about 17 Å, microporous, which is larger than the Hg(II) hydrated radius (2.45 Å) that permits the Hg(II) pore diffusion through the DAC@AHTT pores^72^.

The solubility of DAC@AHTT and DAC@AHTT-Hg(II) was investigated by applying different solvents, including NaOH (0.1–1 mol.L^− 1^), HCl (0.10–1.0 mol.L^− 1^), and ethanol 99.9%. It was considered that the DAC@AHTT and DAC@AHTT-Hg(II) are not soluble in any of the utilized solvents.

**Table 3:** Surface area of native cellulose and DAC@AHTT adsorbent.

Sample	S_BET_, m^2^.g^− 1^	Pore diameter (nm)	Reference
Native cellulose	10.4	–	^14^
DAC@AHTT	42.4	1.7	This work

### Characterization

#### SEM

**Fig. 3:**
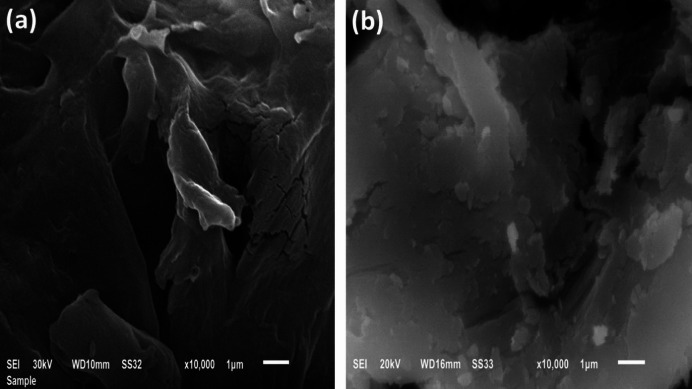
SEM images of (**a**) DAC and (**b**) DAC@AHTT adsorbent.

Surface morphologies of DAC and DAC@AHTT are shown in Fig. 3a and b, respectively. Figure 3a shows that the DAC surface is slightly smooth, fibrous, and exhibits little erosion and some cracks, which may be attributed to the insertion of aldehyde groups following the oxidation step. As presented in Fig. 3b, the DAC@AHTT has more roughness and more compact structure than the DAC material with accumulated granular particles than the DAC material, which may be attributed to the chemical modification by the AHTT ligand. Therefore, these changes enhance the affinity and ability of cellulosic-based adsorbents for the adsorption of metal ions.

#### FTIR spectra

**Fig. 4:**
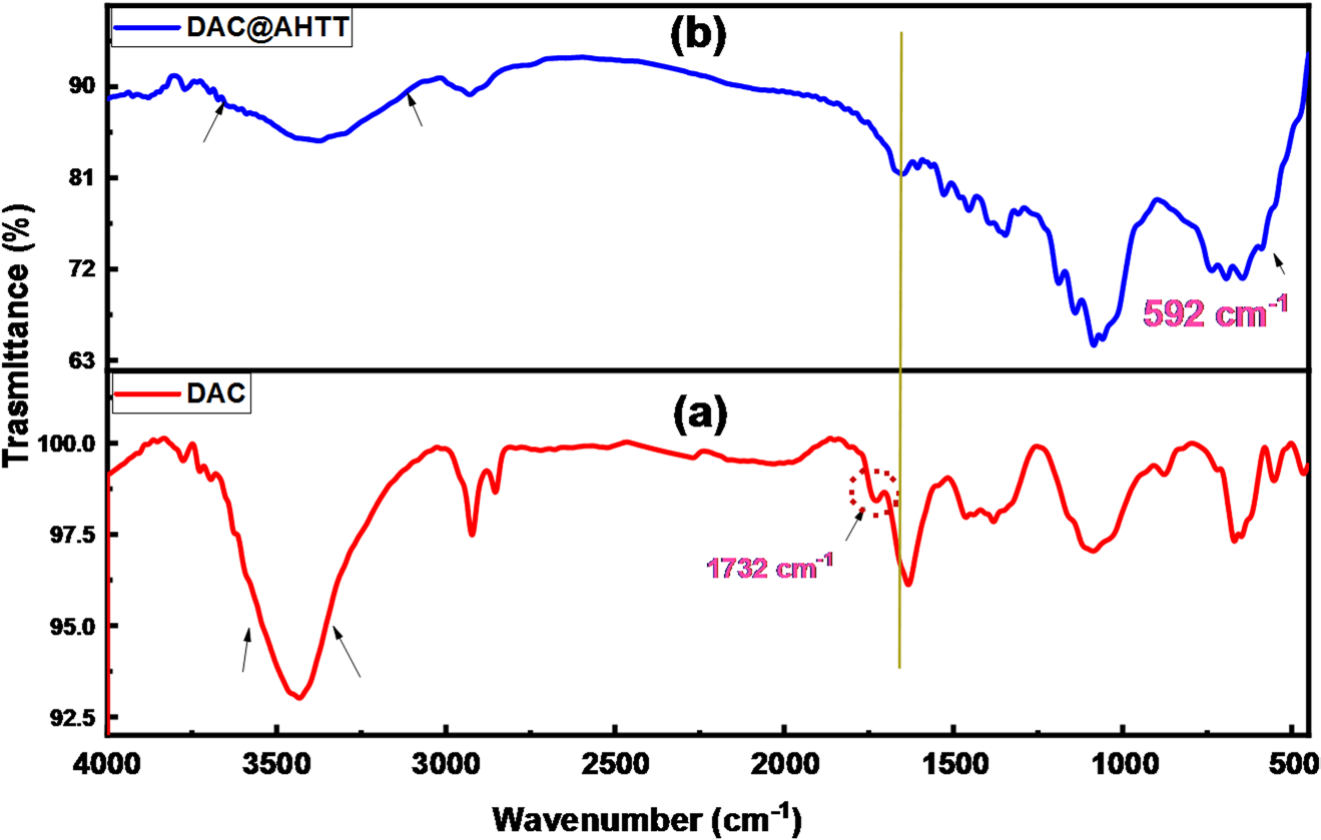
FTIR spectra of (**a**) DAC and (**b**) DAC@AHTT adsorbent.

 Figure 4 shows the FT-IR spectra of the DAC and DAC@AHTT adsorbents. The FT-IR spectrum of DAC, Fig. 4a, displayed various distinguishing bands of the cellulosic materials, including the bands that occurred approximately in the 1000:1200 cm^-1^ and 2700:3000 cm^-1^ ranges, which are attributed to the (C-O) and (C-H) stretching bands, respectively^14^. Besides, those bands that appeared approximately at 3200:3600 and 1260:1410 cm^-1^ are returned to the O-H stretching and bending vibrations, accordingly. Moreover, the characteristic bands of oxidized cellulose, including those of RCHO (aldehyde), appeared at approximately 1732 cm^-1^^14,73^.

The functionalization of the DAC with the AHTT yields some shifts, as it appeared in Fig. 4b. A band at 1678 cm^-1^ was observed, which may be assigned to C = N formation between the AHTT amino groups and the DAC aldehyde groups^14^. Furthermore, the band at 3100:3600 cm^-1^ broadness increased, which may be due to the occurrence of overlapping between the adsorption bands of both hydroxyl and amino groups^50^. Also, the existence of a band at about 592 cm^-1^ may be attributed to the C-S^74^. The FTIR spectra of DAC@AHTT adsorbent and DAC@AHTT-Hg(II) is provided and discussed at the section: mechanism of adsorption.

#### Elemental analysis

Table 4 shows the CHNS results of native cellulose and DAC@AHTT. It was observed that the nitrogen and sulfur contents significantly increased to 17.05% and 5.85%, respectively, after the DAC modification step with the AHTT ligand. Therefore, these indications confirm that DAC@AHTT is successfully formed. The concentration of the inserted AHTT units was calculated to be approximately 4.86 mmol.g^− 1^.

**Table 4:** Elemental analysis of native cellulose and DAC@AHTT materials.

Material	C%	H%	*N*%	S%
Native cellulose	44.5	7.14	0	0
DAC@AHTT	38.4	4.66	17.05	5.85

To fully characterize DAC@AHTT material, various techniques have been employed. The SEM analysis was utilized to evaluate the morphological properties. Moreover, the structural analysis was obtained by applying both CHNS and FT-IR. The pH_PZC_ and S_BET_ were investigated to detect DAC@AHTT surface properties. Table 5 presents a comparison of the characterization techniques used for DAC@AHTT and other previously published materials. It was proven that the current investigation operated a broader and more integrated group of characterization analyses.

**Table 5:** Comparison of the used characterization techniques with other previously published materials.

Sample	Morphological analysis	Structural analysis	Surface properties	Ref
HCM	–	**CHNS**: C = 57.28%, H = 6.39%, *N* = 16.52%, and S = 12.55%. ^**1**^ **HNMR**: 1.35 ppm (CH_3_ × 3), 2 ppm (-NH), 7.21–7.58 ppm (Ph-OH), 8.56 ppm (NH_2_), and 8.74 ppm (CH of benzylidene imine). ^**13**^ **CNMR**: 34.5 ppm ( C aliphatic), 110.8-130.2 ppm (C of Ph-OH), 146 ppm (CH of benzylidene imine), and 178.5 ppm (C of thioamide).	**S**_**BET**_:411 m^2^.g^-1^	^75^
OCM	**TEM** (mesoporous surface)	–	**S**_**BET**_:371 m^2^.g^-1^	^76^
Chitosan-cotton composite	**SEM** (highly rough with irregular grains)	**FT-IR**: 3273 cm^-1^ (OH, NH stretching), 1795 cm^-1^ (C = O stretching), 1380 cm^-1^ (CN axial deformation), and 1040 cm^-1^ (C-O bending).	–	^77^
MeCM	**TEM** (mesoporous and uniform pore size)	–	**S**_**BET**_:573 m^2^.g^-1^	^78,79^
DAC@AHTT	**SEM** (highly rough)	**CHNS**: C = 38.4%, H = 4.66%, *N* = 17.05%, and S = 5.85%. **FT-IR**: 3100–3600 cm^-1^ (OH and NH_2_ overlapping), 1678 cm^-1^ (C = N stretching), and 592 cm^-1^ (C-S).	**S**_**BET**_:42.4 m^2^.g−1 **pH**_**PZC**_ **=** 4.9	This work

### Adsorption studies of Hg(II) in a single-component system

#### Point of zero charge (pH_PZC_)

**Fig. 5:**
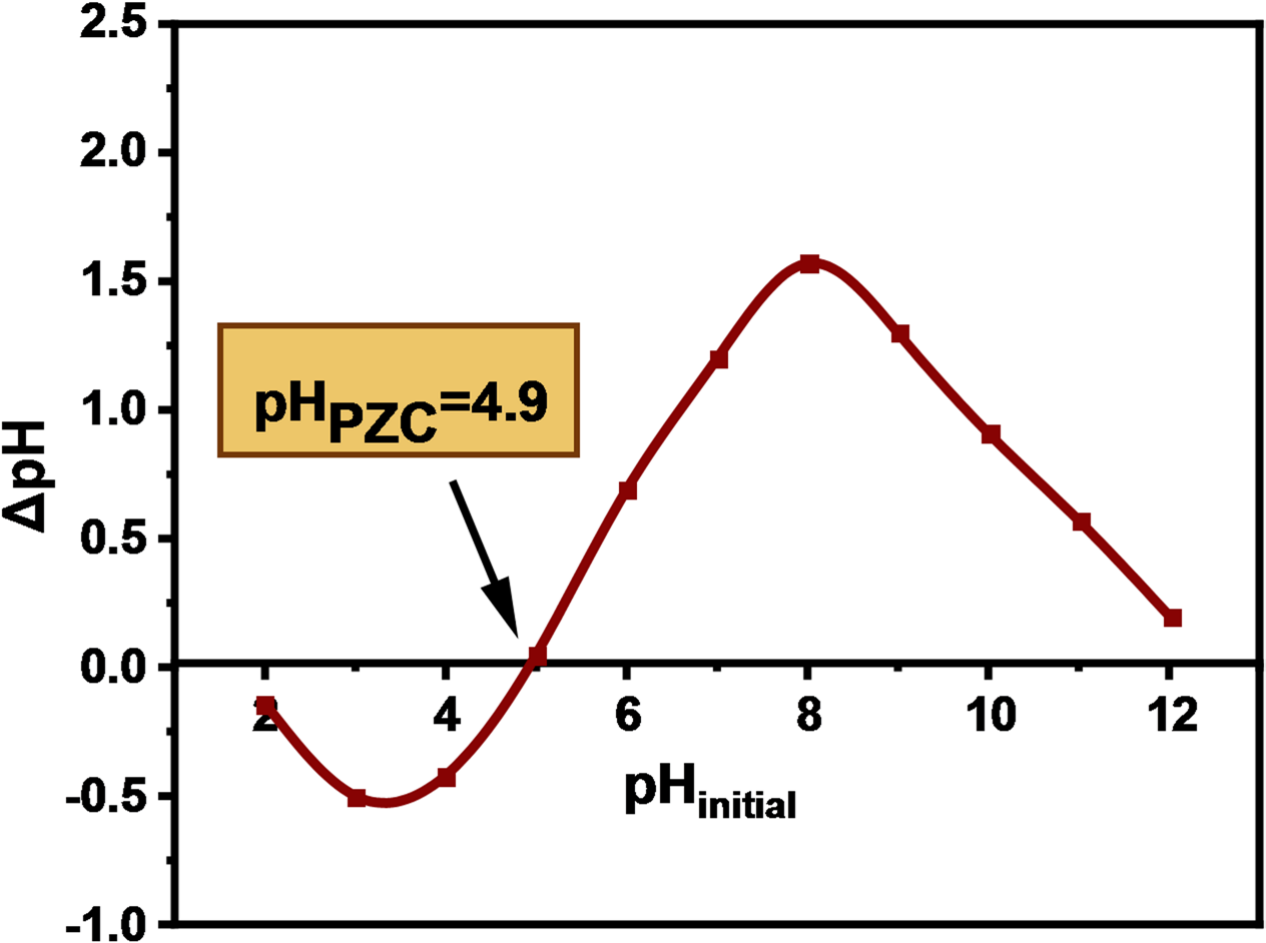
pH_PZC_ of the DAC@AHTT adsorbent.

The pH_PZC_, as shown in Fig. 5, was estimated as follows: the pH value at ΔpH = 0. Generally, DAC@AHTT adsorbent exhibits higher affinities for cations at pH values higher than its pH_PZC_, and vice versa. The pH_PZC_ of DAC@AHTT adsorbent was found to be around 4.7. The Hg(II) adsorption by the DAC@AHTT was expected to be enhanced at a pH value higher than the pH_PZC_ value.

#### Effect of pH

**Fig. 6:**
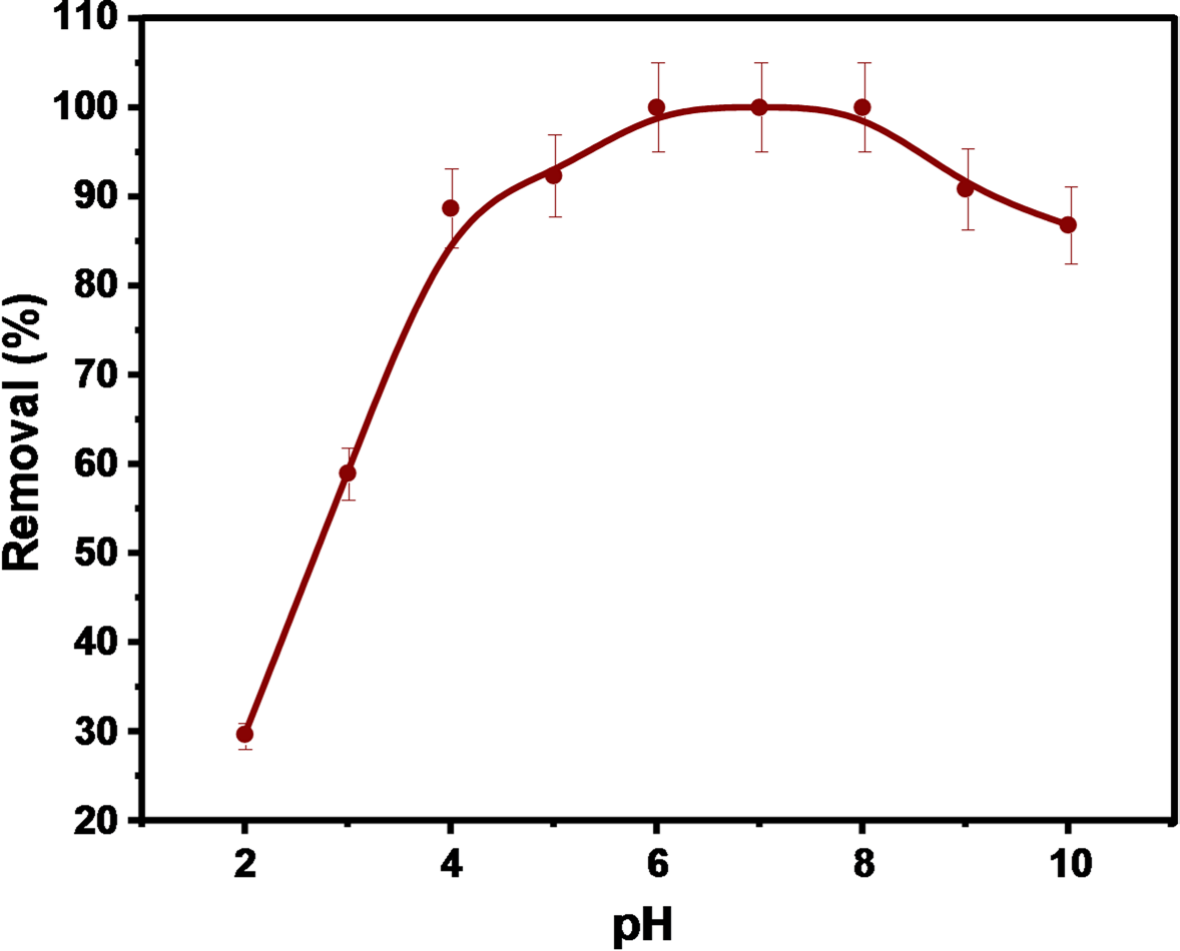
Effect of pH on Hg(II) adsorption onto DAC@AHTT.

As pH can influence the solubility, speciation, and degree of ionization of adsorbents, it is considered an important parameter. In the pH range of 2–10 to avoid the precipitation of the Hg ions in the high alkaline systems, the DAC@AHTT adsorbent adsorption behavior has been investigated. The current experiment was conducted using 0.01 g of the DAC@AHTT adsorbent, which was placed in 10 mL of a 100 mg.L^− 1^ Hg(II) solution for 4 h. As shown in Fig. 6, with the pH increasing from 2 to 6, the Hg(II) adsorption increased from 29.65% to 100%, respectively. Moreover, the adsorption (%) became constant with the elevation of the pH from 6 to 8, then it decreased at a pH value higher than 8.

The Hg(II) predominant species at different pH values are as follows: at pH 2 are HgCl_2_ (63.25%), HgCl^+^ (25.20%), Hg(II) (3.90%), HgOHCl (2%), and HgOH^+^ & Hg(OH)_2_ (in minor quantities), at pH 4 are Hg(OH)_2_ (39.90%), HgOHCl (25.20%), the HgCl_2_ (10.02%), and a small percents of HgOH^+^, HgCl^+^, and Hg(II). At a pH from 6 to 8, the Hg(OH)_2_ (79.62%) and HgOHCl (10.02%)^80–82^. The higher DAC@AHTT affinity toward Hg(II) at elevated pH values may be attributed to the fact that the DAC@AHTT adsorbent contains SH groups that are considered soft bases. At the same time, Hg(II) ions are soft acids. Following the theory of Pearson, throughout the reaction of acids and bases, a hard acid interacts with the hard base, while a soft acid coordinates with the soft base. Neutral molecules are supposed to be softer acids.

From the results presented in Fig. 6, it is evident that Hg(II) adsorption utilizing DAC@AHTT is primarily dependent on the solution pH. The adsorption of Hg(II) utilizing cellulose-based materials in response to pH changes has been studied by numerous researchers. Mostafa et al.^14^ investigated the Hg(II) adsorption utilizing DAC@CAH@SK_2_ composite, which is a cellulosic-based material and has pH_pzc_ equal to 6.85 in the pH range of 2–10, and showed a low adsorption % at pH of 2 which increased gradually from pH of 2 to pH of 5 and remained constant from pH of 5 to 8, then decreased with elevating pH more than 8. Bisla et al.^83^ studied the Hg(II) adsorption and removal in the pH range 2–10 utilizing adsorbent, with pH_PZC_ of 7.8, prepared from the cellulose nanofibers that modified with the l-methionine and declared the low Hg(II) adsorption capacity at a low pH value of 2, then achieved a constant adsorption capacity increase with the pH increasing from 4 to 8, and followed by a slight reduction at a pH value of 10. Arias et al.^84^ investigated the Hg(II) adsorption % utilizing lignocellulosic materials, which was low at a pH of 2 and then increased relatively up to a pH of 9. Anirudhan and Shainy^85^ examined the adsorption of Hg(II) in the pH range of 3–11 using a 2-mercapto-benzamide functionalized cellulosic composite that has a pH_PZC_ value of 6.5. It was indicated that the adsorption increased with the pH value from 3 to 8 and then declined sharply as the pH value increased to 11. These studies recommend that the use of cellulose-based adsorbents for adsorbing Hg(II) relies on the properties of the utilized adsorbent, including Hg(II) speciation, pH_PZC_, and functional groups.

#### Effect of DAC@AHTT adsorbent dose

**Fig. 7:**
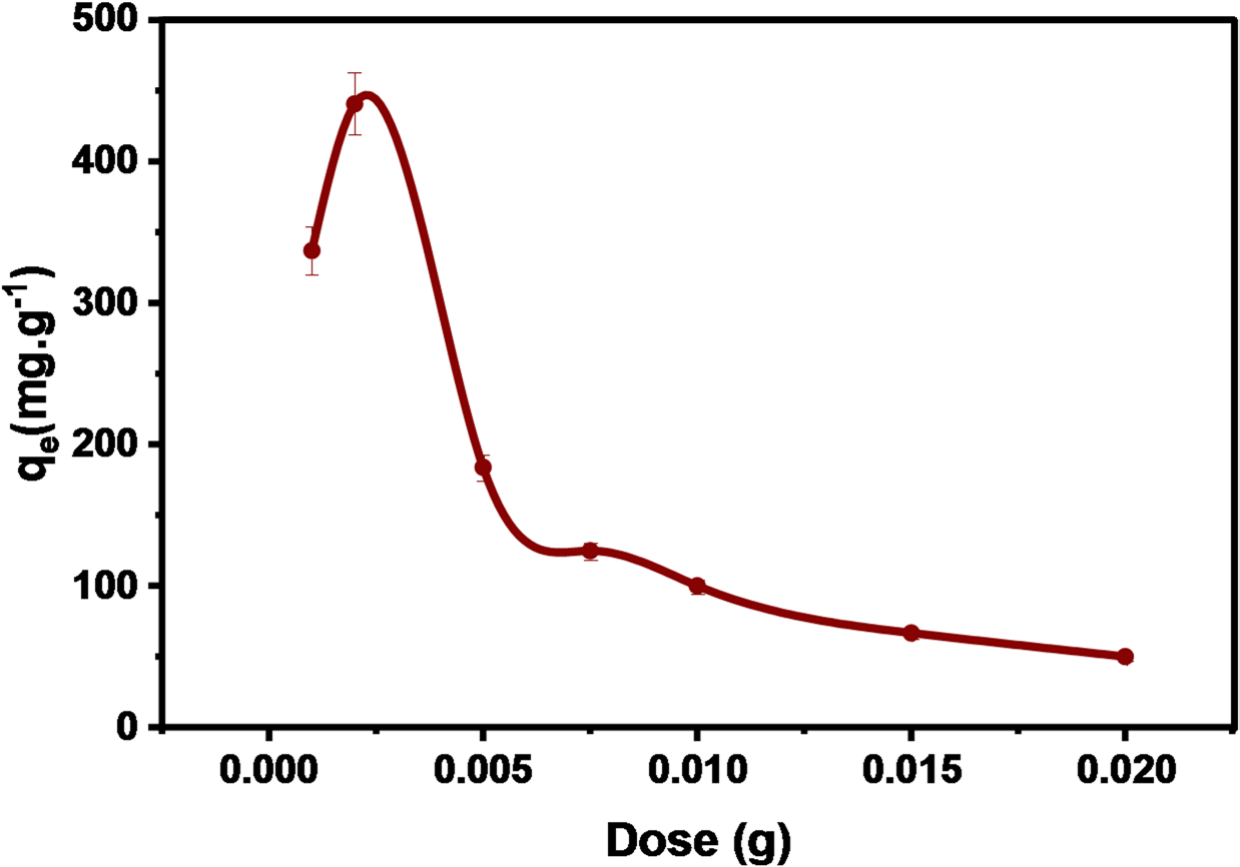
Effect of dose of DAC@AHTT adsorbent on adsorption of Hg(II) (conditions: 10 mL aqueous solution of 10 mg.L^− 1^ for Hg(II) for 240 min at pH 6).

The influence of the dose of DAC@AHTT adsorbent on the Hg(II) adsorption capacity (mg.g^− 1^), Fig. 7, was investigated by applying various doses of DAC@AHTT adsorbent in the range of (0.001–0.002) g. The adsorption capacity of Hg(II) increased from 337 mg.g^− 1^ to 440.75 mg.g^− 1^ by increasing the dose of DAC@AHTT adsorbent from 0.001 g to 0.002 g, respectively. It was observed that increasing the dose of DAC@AHTT beyond 0.002 g resulted in a decrease in capacity to 50 mg.g^− 1^ at a dose of 0.02 g. This may be because DAC@AHTT has no more available surface area and active adsorption sites^14^.

#### Effect of initial concentration and adsorption isotherm

**Fig. 8:**
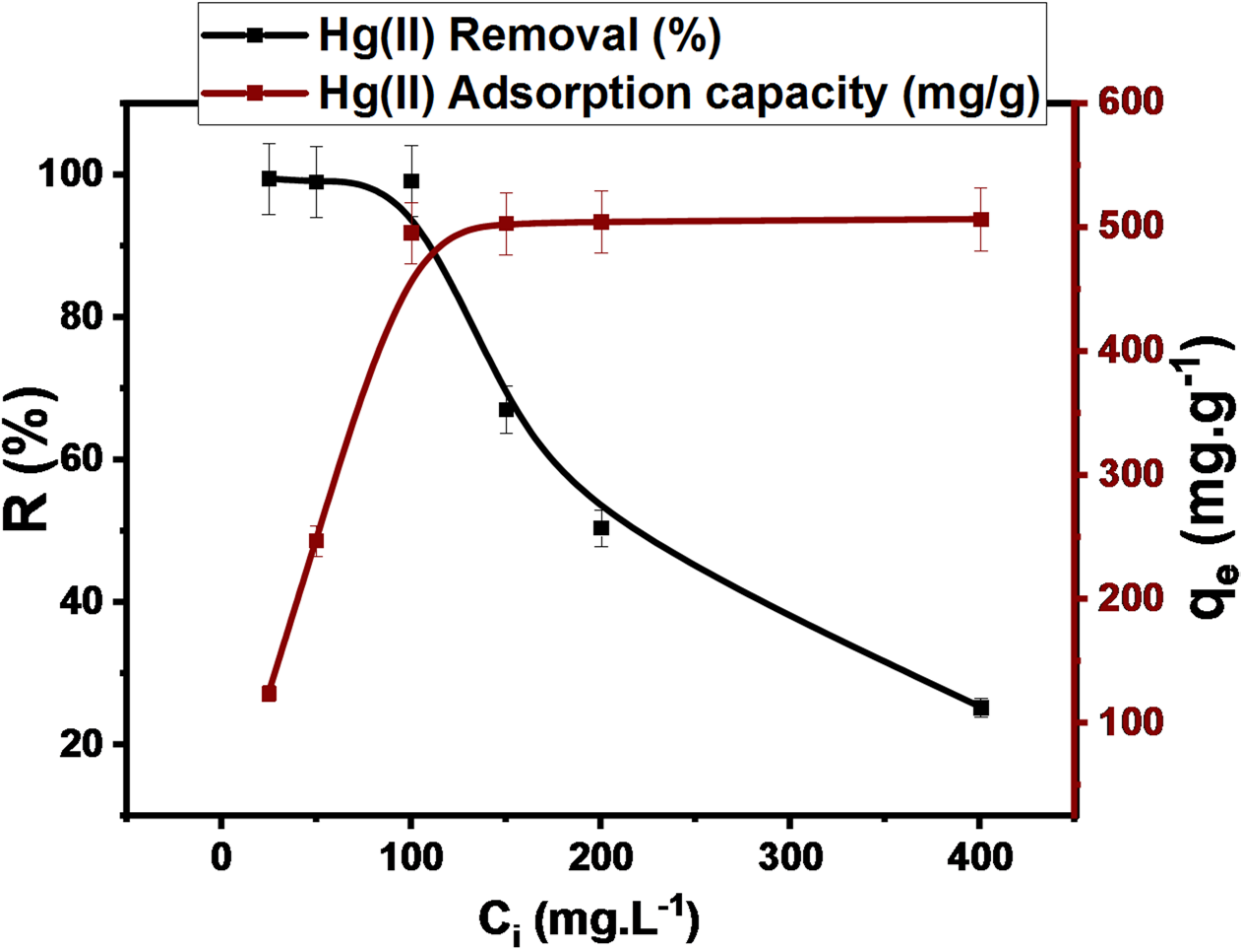
Effect of Hg(II) initial concentration (conditions: 0.002 g of DAC@AHTT was taken at pH 6 for 240 min in the range 25–400 mg.L^− 1^ of Hg(II)).

To evaluate the influence of Hg(II) concentration, which was investigated in the range of 25–400 mg.L^− 1^, on the adsorption efficiency of DAC@AHTT adsorbent, 10 mL adjusted pH solution at 6 of Hg(II) utilizing 0.002 g of DAC@AHTT as a constant amount for 4 h. Figure 8 shows that the interrelated q_e_ (mg.g^− 1^) and R (%) were obtained. It was observed that the DAC@AHTT adsorption capacity increased from 124.3 mg.g^− 1^ to 495.75 mg.g^− 1^ by increasing the concentration from 25 mg.L^− 1^ to 100 mg.L^− 1^. Furthermore, with the increase in Hg(II) concentration from 100 to 400 mg.L^− 1^, the DAC@AHTT capacity tends to stabilize. It was observed that the Hg(II) ion’s q_e_ was increased with Hg(II) initial concentration and reached a maximum to obtain the maximum adsorption capacity. The adsorption amount remains steady and returns to the saturation of the available active sites^86^.

**Fig. 9:**
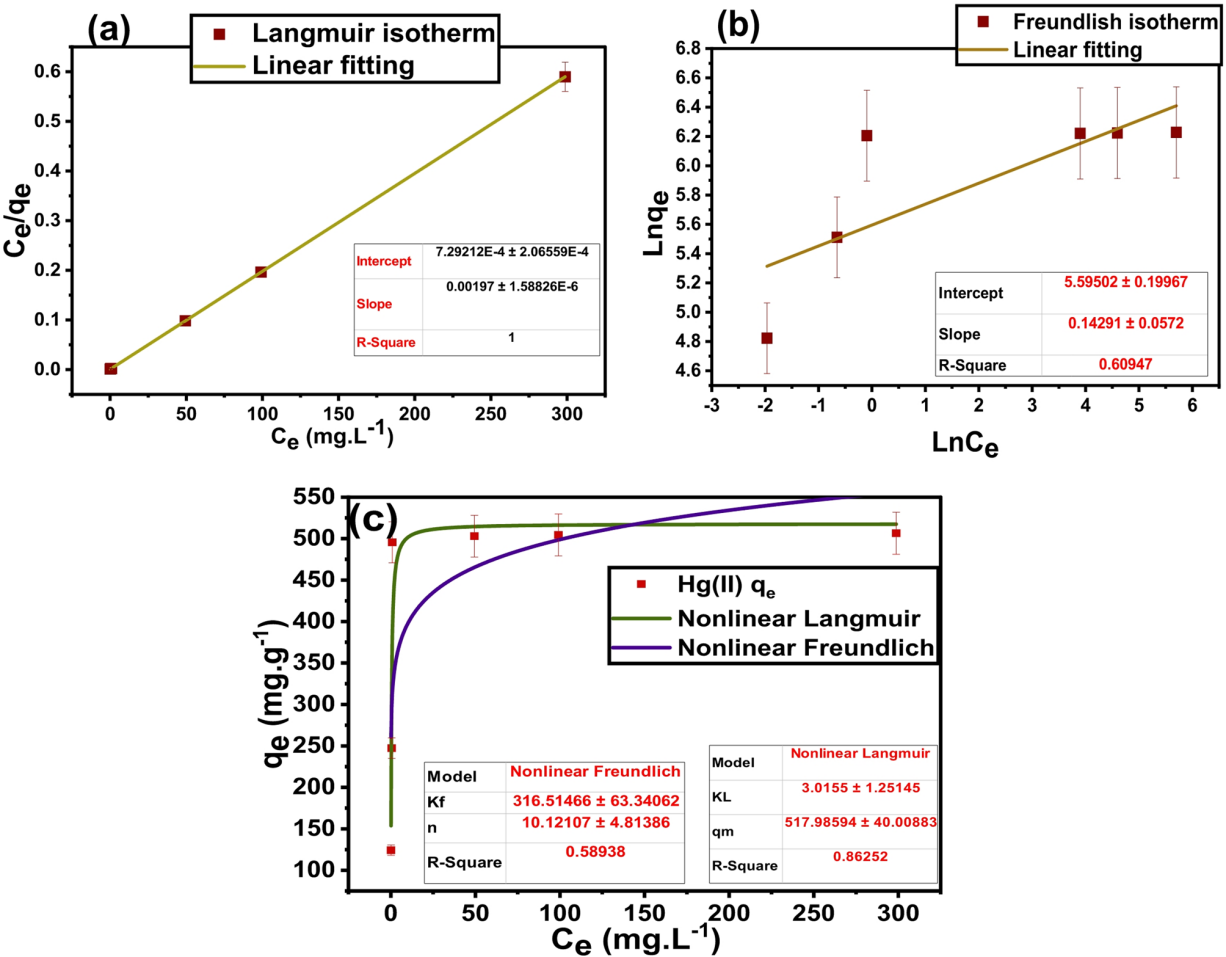
Adsorption isotherms for Hg(II) adsorption by DAC@AHTT: (**a**) Linear Langmuir isotherm model, (**b**) linear Freundlich isotherm model, and (**c**) nonlinear Langmuir and Freundlich isotherm models.

 Figure 9 shows the determined Langmuir and Freundlich isotherms, using both nonlinear and linear methods, for Hg(II) adsorption utilizing DAC@AHTT, and their derived parameters (K_L1_, K_L2,_ K_f1,_ K_f2_, n, and q_m_) are presented in Table 6. From the results in Table 6, it was shown that the Hg(II) adsorption process using DAC@AHTT is chemisorption and follows the linear Langmuir isotherm model as it has the higher R^2^ (1) value and lower χ^2^, SSE, MSE, and hybrid error function values than those of the nonlinear Langmuir as well as linear and nonlinear Freundlich model^30^.

**Table 6:** Adsorption isotherm parameters of Hg(II) by DAC@AHTT adsorbent.

Material	**Linear Langmuir isotherm constants**						
	K_L1_ (L/g)	q_m1_(mg.g^− 1^)	R^2^	χ^2^	SSE	MSE	Hybrid
DAC@AHTT-Hg(II)	2.7	507.6142	1	423.2	2.15*10^5^	3.58*10^4^	-106.836
	**Linear Freundlich isotherm constants**						
	K_F1_	N	R^2^	χ^2^	SSE	MSE	Hybrid
	1.15363	0.17873	0.60947	1.59*10^6^	1.08*10^6^	1.8*10^5^	419.3
	**Nonlinear Langmuir isotherm constants**						
	K_L2_ (L/g)	q_m2_(mg.g^− 1^)	R^2^	χ^2^			
	3.0155	517.98	0.865	442.57			
	**Nonlinear Freundlich isotherm constants**						
	K_F2_	n	R^2^	χ^2^			
	316.5	10.12	0.589	583.15			

#### Effect of oscillating time and adsorption kinetics

**Fig. 10:**
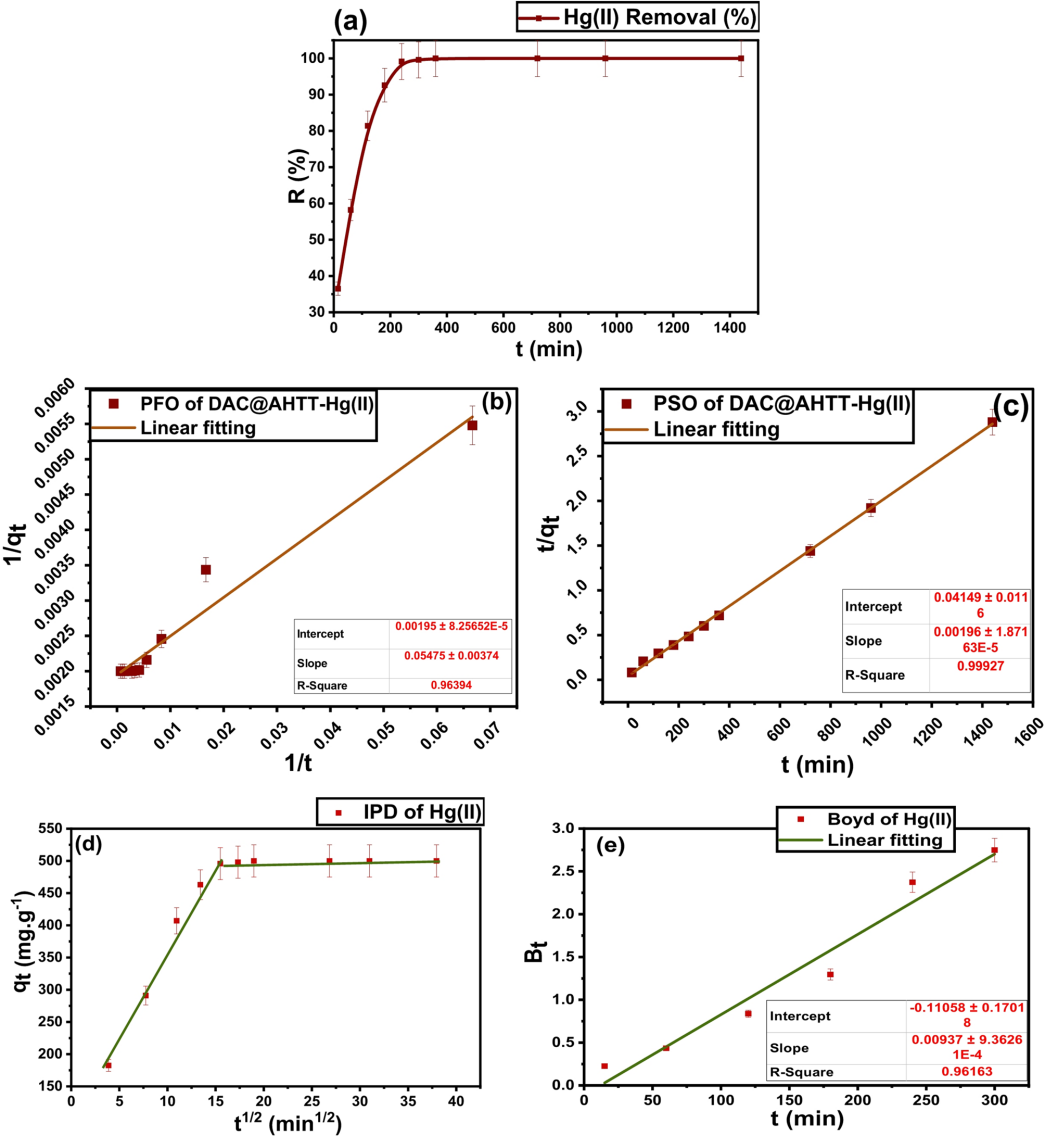
(**a**) Effect of oscillation time on adsorption of Hg(II) (conditions: 0.002 g of DAC@AHTT, 10 mL of 100 mg.L^− 1^ Hg(II), Temp.: 25 °C, time: 15–1440 min), (**b**) PFO kinetic model for Hg(II) adsorption, (**c**) PSO kinetic model for Hg(II) adsorption, (**d**) IPD kinetic model for Hg(II) adsorption, and (**e**) Boyd kinetic model for Hg(II) adsorption.

The kinetic investigation was conducted by studying the influence of oscillating time, as shown in Fig. 10a, in the range of 15–1440 min using 0.002 g of DAC@AHTT, which was added to 10 mL of a 100 mg.L^− 1^ Hg(II) solution. It is observed that the adsorption capacity of DAC@AHTT increases with the increase in oscillating time from 15 min to 240 min, reaching a q_e_ value of 495.75 mg.g^− 1^. By increasing the oscillating time by more than 240 min, the DAC@AHTT adsorption capacity became constant, and the Hg(I) adsorption attained equilibrium.

 Figure 10b,c illustrates the experimental data fitting to the Pseudo-1st -order (PFO) and Pseudo-2nd -order (PSO) models. Moreover, the derived kinetic parameters (K_1_, K_2_, q_e1ads_, q_e2ads_, and R^2^) are exhibited in Table 7. The Hg(II) adsorption utilizing DAC@AHTT achieved equilibrium within 240 min (Fig. 10a). The correlation coefficients (R^2^ = 0.999) corresponding to the PSO for both studied dyes are larger than those for the PFO. Therefore, the calculated q_e_ value (510.2 mg.g^− 1^) from the PSO for Hg(II) is highly comparable to that obtained from experiments (497.45 mg.g^− 1^). This demonstrates that the PSO kinetic model was well-matched to the experimental data for Hg(II) adsorption^32^. This may be attributed to the fact that at the beginning, the functional groups were abundant and a high concentration of the pollutant accumulated onto the adsorbent surface. Active sites have S and N donor groups that are strongly bound to the metal ions. Afterwards, the availability of adsorption active sites decreases, resulting in slower adsorption^87,88^.

The Hg(II) adsorption multi-linearity process is observed in Fig. 10d, suggesting that two stages are involved in the adsorption process. Hg(II) external diffusion onto the DAC@AHTT was responsible for the first linear step, while delayed intra-particle Hg(II) diffusion was responsible for the second linear step. The deviation from the origin (C ≠ 0) confirms that the intraparticle diffusion is not the sole rate-limiting step, but it contributes to film diffusion. The Boyd plot, Fig. 10e, shows a very small intercept near zero, indicating that the rate-controlling mechanism for the Hg(II) adsorption process on DAC@AHTT is intraparticle diffusion.

**Table 7:** Kinetic parameters for the adsorption of Hg(II) by DAC@AHTT.

Material	**PFO kinetic model**						
	K_1_ (min^− 1^)	q_e1ads_(mg.g^− 1^)	R^2^	χ^2^	SSE	MSE	HYBRID
DAC@AHTT-Hg(II)	28.07692	512.82	0.95943	337.67	173163.75	17316.37	34.5
	**PSO kinetic model**						
	k_2_ (g/(mg min))	q_e2ads_ (mg.g^− 1^)	R^2^	χ^2^	SSE	MSE	HYBRID
	9.2591*10^− 5^	510.2	0.99918	331.41	169087.81	16908.78	33.77
	**IPD kinetic model**						
	k_int_ (mg.(g. min^1/2^))^**−1**^		c (mg.g^− 1^)		R^2^		
	7.5		296		0.5223		
	**Boyd’s kinetic model**						
	Intercept			R^2^			
	-0.11			0.96163			

#### Thermodynamics

**Fig. 11:**
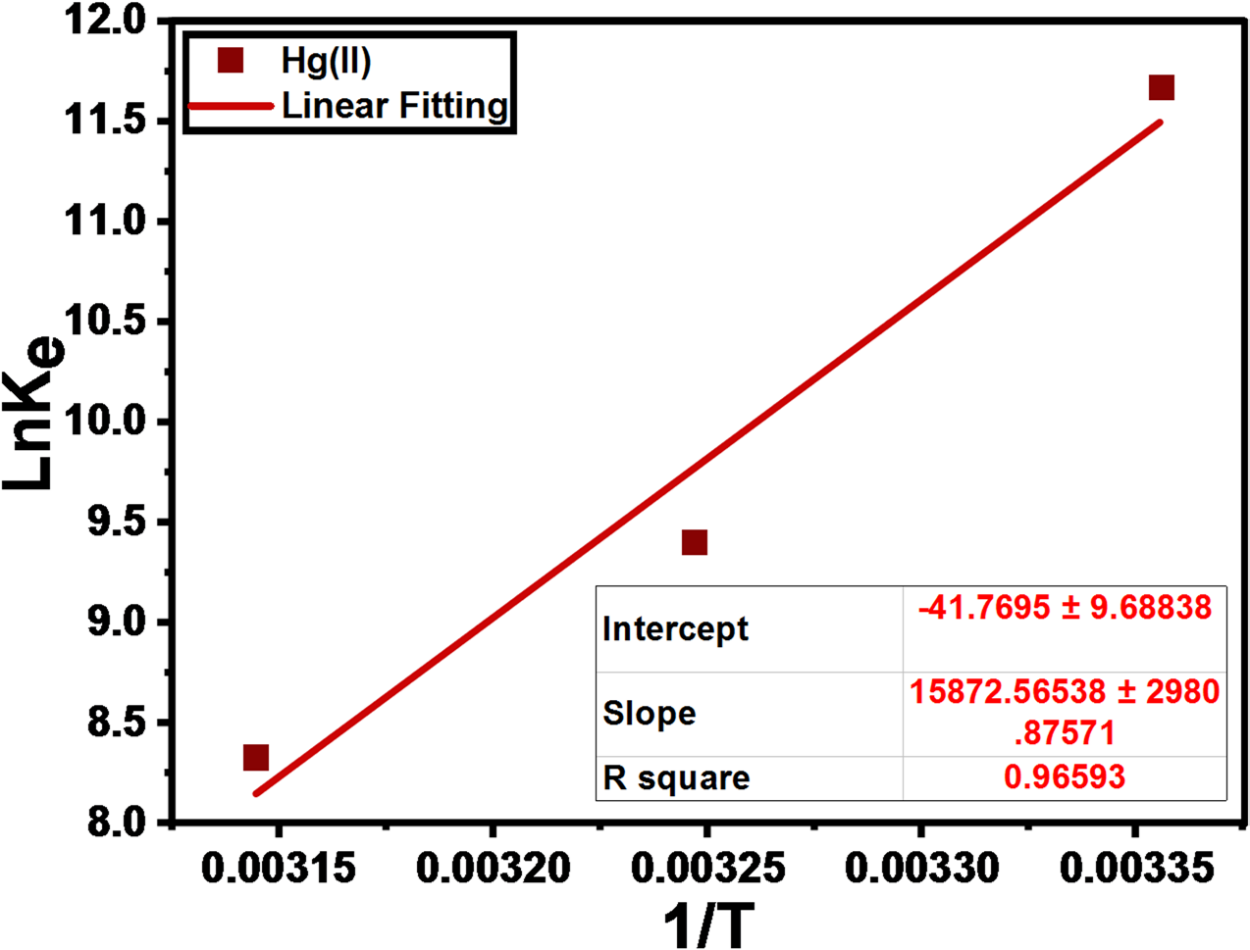
Plot of lnK_e_ vs. (1/T) absolute temperature for the adsorption of Hg(II).

The ΔH^o^_ads_ and ΔS^o^_ads_ values were estimated from the slope and intercept, respectively, from Fig. 11. As presented in Table 8, the estimated negative ΔG^o^_adsn_ values reveal that the Hg(II) adsorption by DAC@AHTT adsorbent is spontaneous, in addition to the thermodynamic feasibility of the Hg(II) adsorption under the investigated temperature range. Moreover, the ΔH^o^_ads_ negative charge and its value (131.96 kJ.mol^− 1^) indicate that the Hg(II) adsorption process was exothermic and chemisorption involving the formation of Hg–S and Hg–N coordination bonds between Hg(II) ions and the functional groups of DAC@AHTT, respectively. Additionally, the arrangement-increasing and disorder-reducing phenomenon was demonstrated by the negative value of ΔS^o^_ads_, which may result from the orderly attachment of Hg(II) ions onto specific active sites, thereby replacing more mobile water molecules. These findings together confirm that Hg(II) removal proceeds through an energetically favored chemisorption pathway^85^.

**Table 8:** Thermodynamic parameters for the adsorption of Hg(II) onto DAC@AHTT.

System	T (k)	K_c_	ΔG^o^_ads_ (KJ.mol^− 1^)	ΔH^o^_ads_ (KJ.mol^− 1^)	ΔS^o^_ads_ (J.mol^− 1^.K^− 1^)
DAC@AHTT-Hg(II)	298	583.24	-15.7786	-131.96	-347.27
	308	60.36	-10.4997		
	318	20.71	-8.01211		

#### Ionic strength

**Fig. 12:**
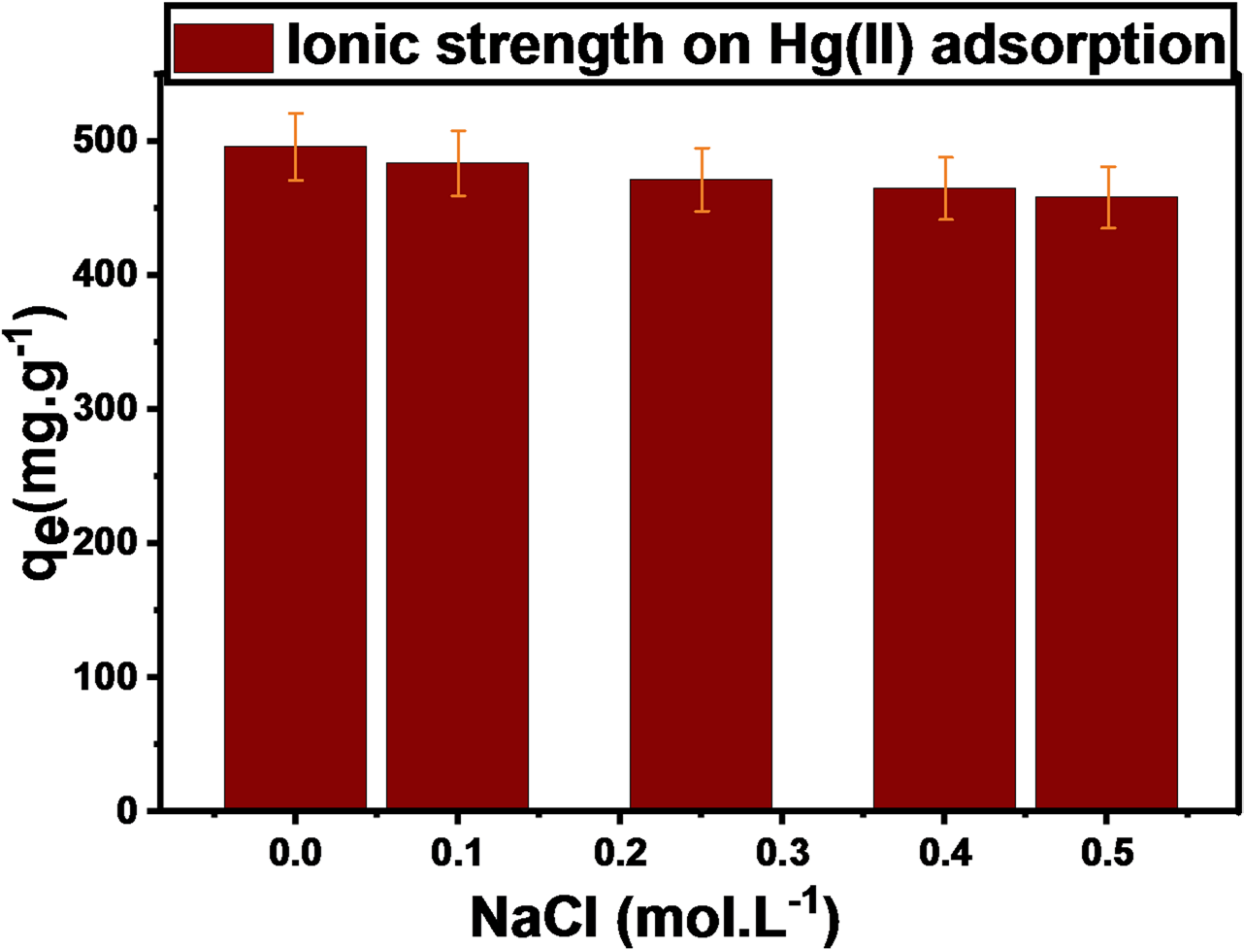
Effect of NaCl ionic strength on the Hg(II) adsorption (conditions: 0.002 g of DAC@AHTT was taken at 10 mL 100 mg.L^− 1^ of Hg(II) at pH 6 for 240 min).

The parameter of ionic strength was investigated by applying various inorganic electrolyte concentrations (Cl^−^) in the form of NaCl. The investigation utilized 0.002 g of DAC@AHTT, which was added to a 10 mL aqueous solution containing 100 mg.L^− 1^ of Hg(II), at 25 °C for 240 min, with an electrolyte concentration range of (0 mol.L^− 1^- 1 mol.L^− 1^). From the findings shown in Fig. 12, it was concluded that the DAC@AHTT adsorbent’s adsorption capacity for Hg(II) decreased with the investigated Cl^−^ inorganic electrolyte. As the presence of Cl^−^ groups results in Hg(II) affinity to SH and NH_2_ groups decreasing. This is attributed to the fact that Hg(II) has a high affinity to form stable complexes, including HgCl_2_, HgCl_3_^−^, and HgCl_4_^−−^. These complexes have high solubility and low affinity toward SH and NH_2_ functional groups. Different sulfur- and nitrogen-containing sorbents have been shown to have similar effects on Hg(II) adsorption when chloride ions are present^89–91^.

#### Desorption and regeneration

Adsorbent regeneration and reuse are important parameters for potential real wastewater treatment approaches. Ligand-based adsorbents are highly reusable and retain nearly their original functionality for metal ion capture^92^. For further reusing, various eluents were tested for the Hg(II) desorption from the DAC@AHTT as ethanol (99.9%), HCl (0.1 mol.L^− 1^-0.3 mol.L^− 1^), NaOH (0.05 mol.L^− 1^), NaHCO_3_ (0.1 mol.L^− 1^-0.3 mol.L^− 1^), thiourea (0.05 mol.L^− 1^-0.2 mol.L^− 1^), HNO_3_ (0.1 mol.L^− 1^-0.3 mol.L^− 1^), and (1:1) mixture of 0.1 mol.L^− 1^ thiourea and 0.1 mol.L^− 1^ HNO_3_. It was observed that the thiourea/HNO_3_ mixture was the most effective eluent among all investigated eluents and was successfully used for the desorption of the Hg(II) from DAC@AHTT. The DAC@AHTT adsorbent has good recycling performance. After undergoing three regeneration and adsorption cycles, the material still exhibits more than 85% Hg(II) removal efficiency, as presented in Table 9, indicating its high structural stability. It was anticipated that DAC@AHTT adsorbent could be a good and efficient sorbent for Hg(II) removal from aqueous solutions.

**Table 9:** Repeated adsorption-desorption cycles for DAC@AHTT regeneration by using 0.1 mol.L^− 1^ thiourea/0.1 mol.L^− 1^ HNO_3_ mixture (1:1).

Cycle number	Hg(II) in eluate (mg.L^− 1^)	Desorption (%)	Hg(II) concentration (mg.L^− 1^)	Recovery (%)
1	97.2	98.1	4.5	96.37
2	93.145	94	9.035	91.8
3	89.49	90.3	15.65	85.12
4	82.44	83.2	23.29	77.41
5	73.33	74	32.62	68

#### Application

The application experiments of the DAC@AHTT were conducted by adsorbing Hg(II) (50 mg.L^− 1^ and 100 mg.L^− 1^) from tap water, Nile, seawater, and wastewater samples to assess the applicability of DAC@AHTT in real samples. As displayed in Table 10, the Hg(II) recoveries (%) from the experimental real water samples are more than 98%. It was demonstrated that DAC@AHTT exhibits remarkable recoveries for Hg(II) spiked in the tested water samples, proving that DAC@AHTT can be effectively utilized for mercury removal from the aqueous environment in actual practice. To assess the DAC@AHTT sensitivity toward Hg(II) and its applicability in real water, it was applied to Nile water samples spiked with very low Hg(II) concentrations (0.05–0.5 mg.L^− 1^). It was shown that the high recovery (98–100%) % and the very low RSD (%).

**Table 10:** Analysis of spiked Hg(II) in real water samples by DAC@AHTT (*n* = 3).

Wastewater samples type & location	Added (mg.L^− 1^)	Found (mg.L^− 1^)	q_e_ (mg.g^− 1^)	Recovery (%)	RSD (%)
Sea water, Ras El-bar, Daemitta, Egypt)	0.00	0	0	0	0.00
	50.00	0.73	246.35	99.57559	1.2
	100.00	1.45	492.75	99.45504	0.78
Nile water (Mansoura, Dakahlia, Egypt)	0.00	0	0	0	0.00
	0.05	0	0.25	100	0.16
	0.10	0	0.5	100	0.2
	0.50	0.01	2.45	98	0.1
	50.00	1.1	244.5	98.82781	1.6
	100.00	2.8	486	98.09264	1.4
Tap water (Mansoura University, Mansoura, Egypt)	0.00	0	0	0	0.00
	50.00	0.6	247	99.83832	1.2
	100.00	1.23	493.85	99.67706	0.97
Waste water (Sinbellawin sewage station, Dakahlia, Egypt)	0.00	0	0	0	0.00
	50.00	0.94	245.3	99.15117	1.12
	100.00	1.87	490.65	99.03118	1.3

### Mechanism of adsorption

To investigate the Hg(II) possible mechanism on the DAC@AHTT, digital photographs, BET, SEM, and FTIR of the DAC@AHTT and DAC@AHTT-Hg(II), as well as the EDX of DAC@AHTT-Hg(II), were assessed.

#### SEM

**Fig. 13:**
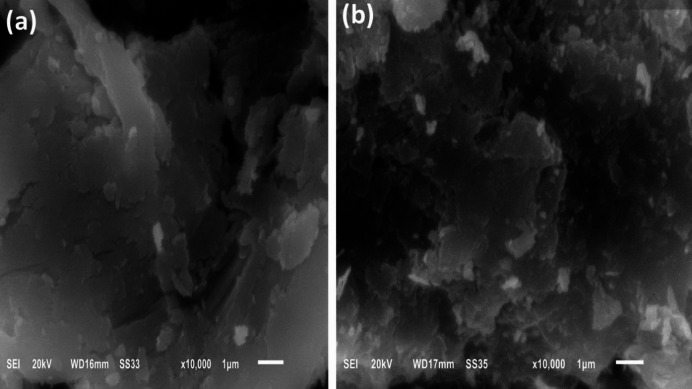
SEM images of (**a**) DAC@AHTT and (**b**) DAC@AHTT-Hg(II).

 Figure 13 presents the DAC@AHTT before and after Hg(II) adsorption. It was observed that the surface became brighter after the Hg(II) adsorption onto the DAC@AHTT. The surface became more compact with pores blocked by the Hg(II) particles. This returns to the reason for the higher electrical conductivity of mercury compared to the DAC@AHTT cellulosic material^14,93^. Moreover, the appearance of clusters on the DAC@AHTT surface may be due to the accumulation of adsorbed Hg(II).

#### EDX

**Fig. 14:**
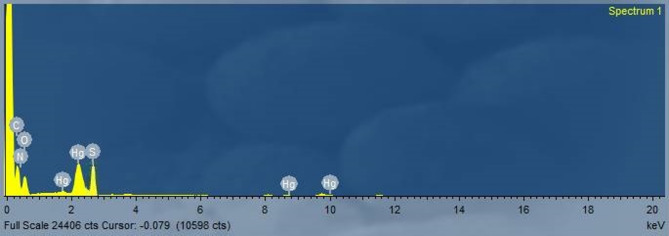
EDX spectral analysis of DAC@AHTT-Hg(II).

The Hg(II) adsorption onto the DAC@AHTT surface was verified and demonstrated through the EDX analysis, as presented in Fig. 14. The current Hg(II) adsorption has been confirmed with the appearance of its characteristic peak between 1 and 3 keV^94^.

#### FTIR spectra of DAC@AHTT adsorbent and DAC@AHTT-Hg(II)

**Fig. 15:**
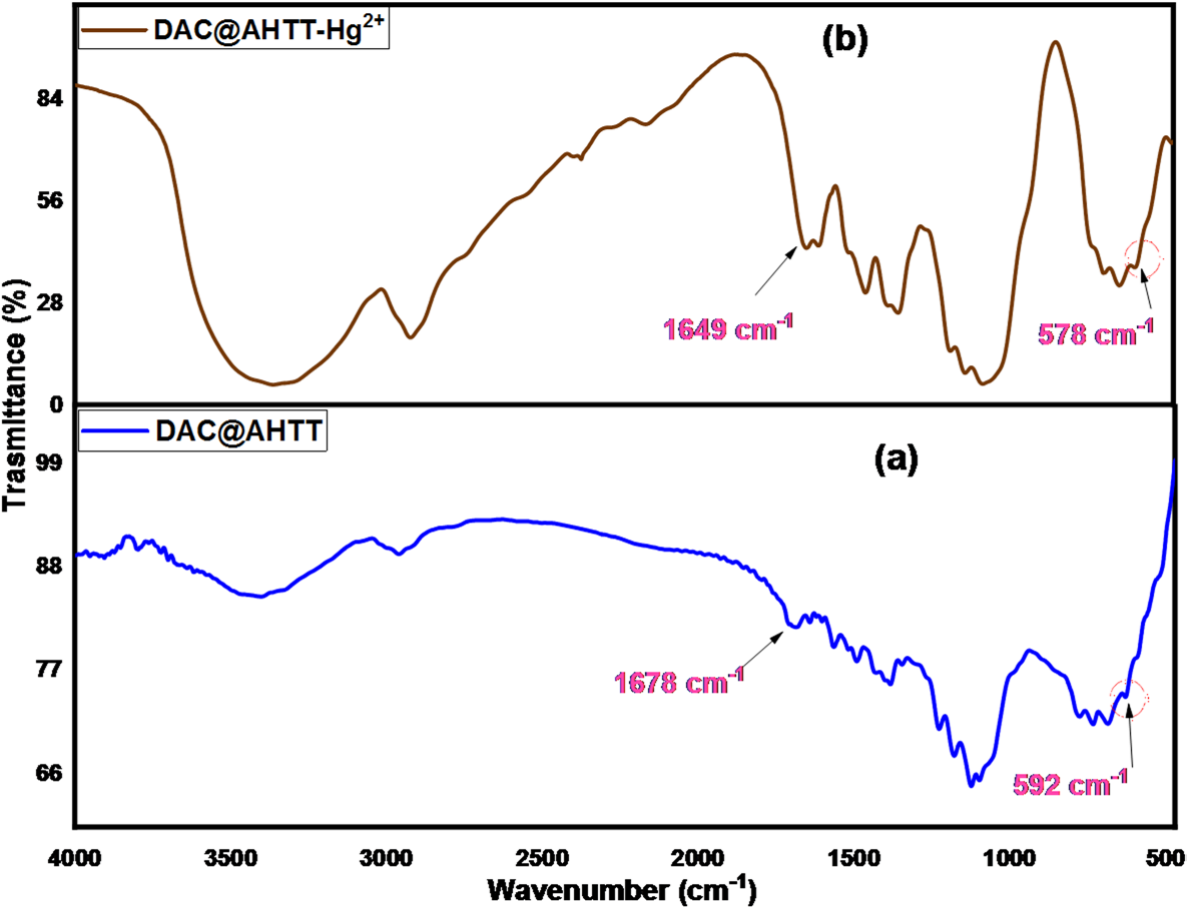
FTIR spectra of (**a**) DAC@AHTT adsorbent and (**b**) DAC@AHTT-Hg(II).

 Figure 15 presents the spectra of the DAC@AHTT adsorbent before and after Hg(II) adsorption. It was noticed that the band of -C = N shifted after the Hg(II) adsorption from 1678 cm^− 1^ (Fig. 15a) to 1649 cm^− 1^ (Fig. 15b). Additionally, the -NH bands also shifted. Furthermore, the DAC@AHTT-Hg(II) spectrum (Fig. 15b) shows that the -C-S group shifted from 592 cm^− 1^ to 578 cm^− 1^ and became broader than that of the DAC@AHTT adsorbent^74^. All previous changes led us to assess that the Hg(II) complexation with the DAC@AHTT may be achieved by forming a Five-ring complex through the -SH and -NH_2_. The Hg(II) complexation may also be carried out through the 3ry amine lone pair and the -NH_2_ group, resulting in a six-ring complex formation.

**Fig. 16:**
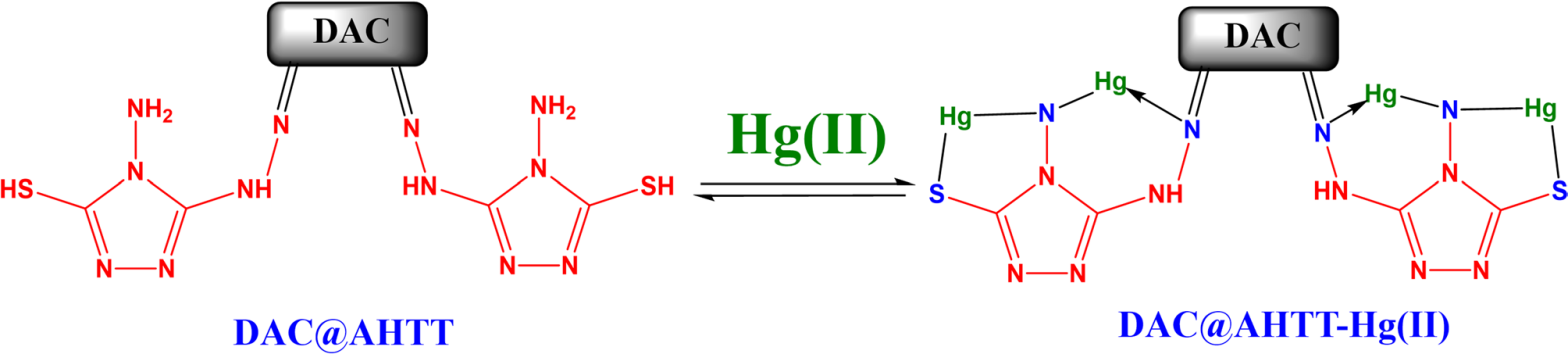
Plausible mechanism of sorption of Hg(II) onto DAC@AHTT adsorbent.

Commonly, the adsorption of metal ions’ plausible mechanism onto any material is either investigated through metal ions exchange with the adsorbent, chelating agent, active groups, or via a chemical reaction between them. The Hg(II) applied mechanisms are chelation, ion exchange, surface complexation, and precipitation for thiol (-SH) functionalized adsorbents, as the Hg(II) empty orbital can be bound with a free SH lone pair of electrons. The current possible mechanism for the Hg(II) adsorption onto the DAC@AHTT adsorbent, according to the analysis discussed above, could be as presented in Fig. 16. The complexation of Hg(II) occurred through the formation of coordination bonds with the DAC@AHTT -S-H, C = N, and -NH_2_, forming two membered rings (five and six), which are considered stable complexes^95,96^.

### Performance of DAC@AHTT adsorbent

To assess the DAC@AHTT adsorbent value, a comparison between DAC@AHTT q_max_, maximum adsorption capacity, and other reported cellulose-based adsorbents for Hg(II) removal was obtained, as presented in Table 11. It was observed that Hg(II) adsorption by the DAC@AHTT is reasonably positioned with respect to other mentioned investigations, with a q_max_ of 495.75 mg.g^− 1^ at 25 °C, as it presents a q_max_ value higher than that of the other reported materials’ q_max_. Compared to other similar thiosemicarbazide modified cellulose adsorbents for Hg(II) removal, it can be noticed that the adsorption capacity DAC@AHTT adsorbent is greater than the adsorption capacity of some similar thiosemicarbazide modified cellulose adsorbents for Hg(II) removal such as Thiosemicarbazide-modified cellulose^97^, Thiocarbohydrazide grafted dialdehyde cellulose nanobiosorbent^98^ and guanyl thiosemicarbazide functionalized dialdehyde cellulose^53^. The difference in capacity between DAC@AHTT adsorbent and other reported cellulose-based adsorbents may be attributed to the morphological properties of each adsorbent, such as structure, active groups, and surface area. There are limited investigations into the removal of Hg(II) using triazole-modified cellulose materials. Magar et al. prepared a composite material consisting of reduced graphene oxide (RGO) conjugated to cellulose-triazole, which was successfully used for Hg(II) detection^99^. However, rather than concentrating on adsorption applications, the majority of current triazole-modified cellulose materials focus on sensing. Thus, creating a dual-functionalized cellulose composite with both triazole and thiol functionalities, like DAC@AHTT, provides a unique method that combines improved surface reactivity and recyclability with a strong affinity for Hg(II) binding. It can be concluded that DAC@AHTT can be utilized as an efficient adsorbent for Hg(II) adsorption. Due to environmental considerations and development requirements, the desorption process and the regeneration of the investigated adsorbent are crucial aspects for evaluating the reusability of adsorbents in industrial applications.

**Table 11:** Comparison of equilibrium time and adsorption capacity of various cellulose-based adsorbents for Hg(II).

Adsorbent	Adsorption capacity (mg.g^− 1^)	Equilibrium time (min)	Optimal pH	Ref.
Thiosemicarbazide-modified cellulose	331.1	100	5	^97^
Thiocarbohydrazide grafted dialdehyde cellulose nanobiosorbent	190	120	6	^98^
Guanyl thiosemicarbazide functionalized dialdehyde cellulose	94	240	6	^53^
Algal biomass	42	90	6	^100^
Waste activated sludge (WAS)	477	120	5	^101^
Polyacrylate-modified carbon (PAMC) composite	76.3	90	6	^102^
Activated Carbon Prepared from Rice Husk	55.87	60	5	^103^
Adulsa (*Justicia Adhatoda)* leaves powder	107.5	40	6	^104^
Charred xanthated sugarcane bagasse (CXSB)	333.34	120	4.5	^105^
Charred sugarcane bagasse (CSB)	125			
Carbamoylethylated Wood Pulp	239.8	60	5	^106^
Sulfurized wood biochar	108	30	6	^107^
Tannic acid cross-linking cellulose/polyethyleneimine functionalized magnetic composite (MCP)	247.51	180	5	^108^
DAC@CAH@SK_2_	139.6	180	5–8	^14^
DAC@AHTT	495.75	240	6–8	This work

### Heavy metal removal from a multicomponent system

#### Digital photographs

**Fig. 17:**
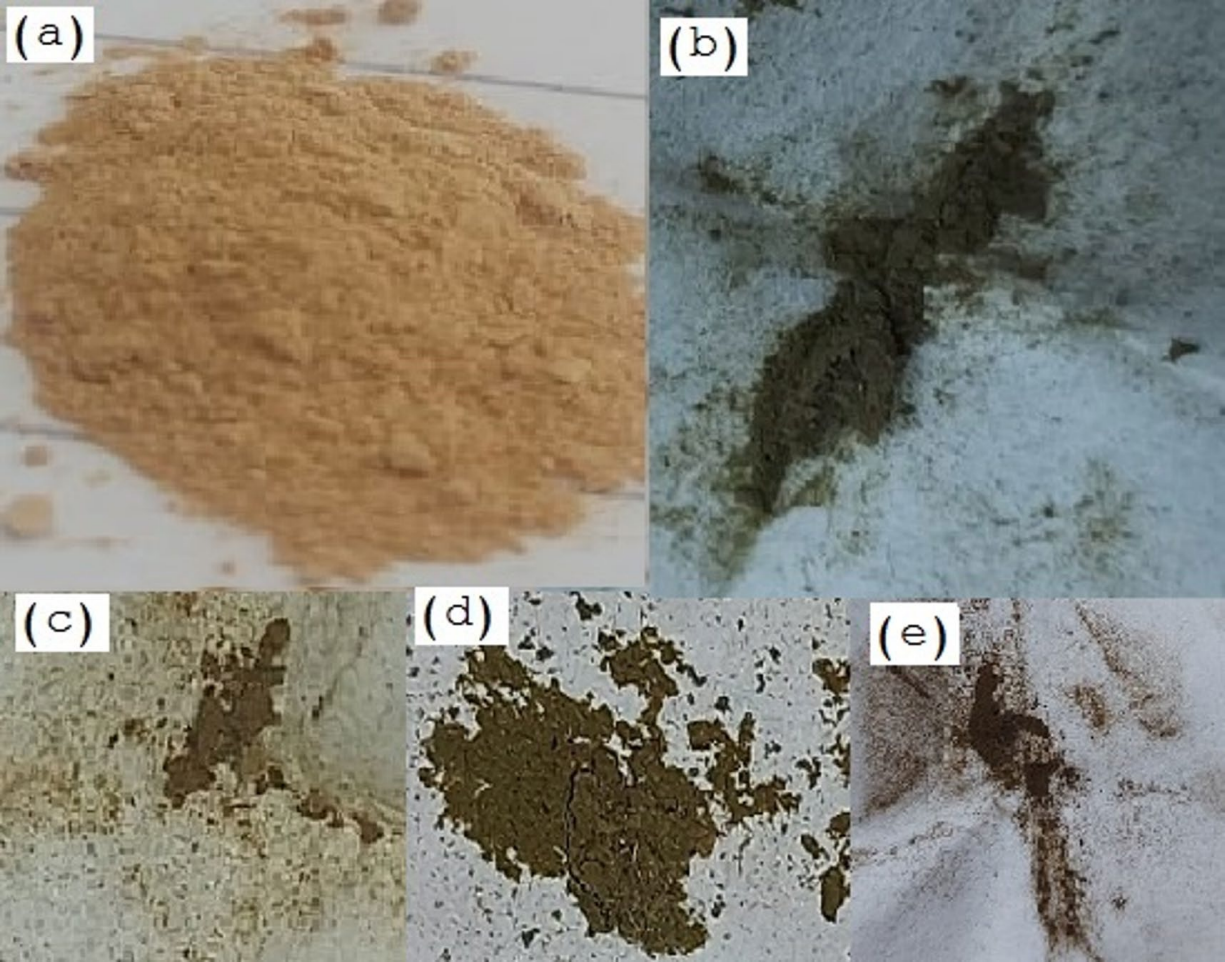
Digital photographs of (**a**) DAC@AHTT adsorbent befor adsorption of metal ions, (**b**) DAC@AHTT**-**Hg(II), (**c**) DAC@AHTT-Ni(II), (**d**) DAC@AHTT-Pb(II), and (**e**) DAC@AHTT-Cu(II).

Noticeable color changes after metal ions adsorption were clearly noticed as it can be noticed in Fig. 17. The color of DAC@AHTT adsorbent is changed from the sandy fawn color (Fig. 17a) to muddy brown (Fig. 17b), olive brown (Fig. 17c), earthy brown (Fig. 17d), and reddish brown (Fig. 17e) following the Hg(II), Ni(II), Pb(II), and Cu(II) adsorption, respectively. These color alterations demonstrated the ability and efficiency of the DAC@AHTT towards various metal ions.

#### FTIR spectra of heavy metals loaded DAC@AHTT adsorbent

**Fig. 18:**
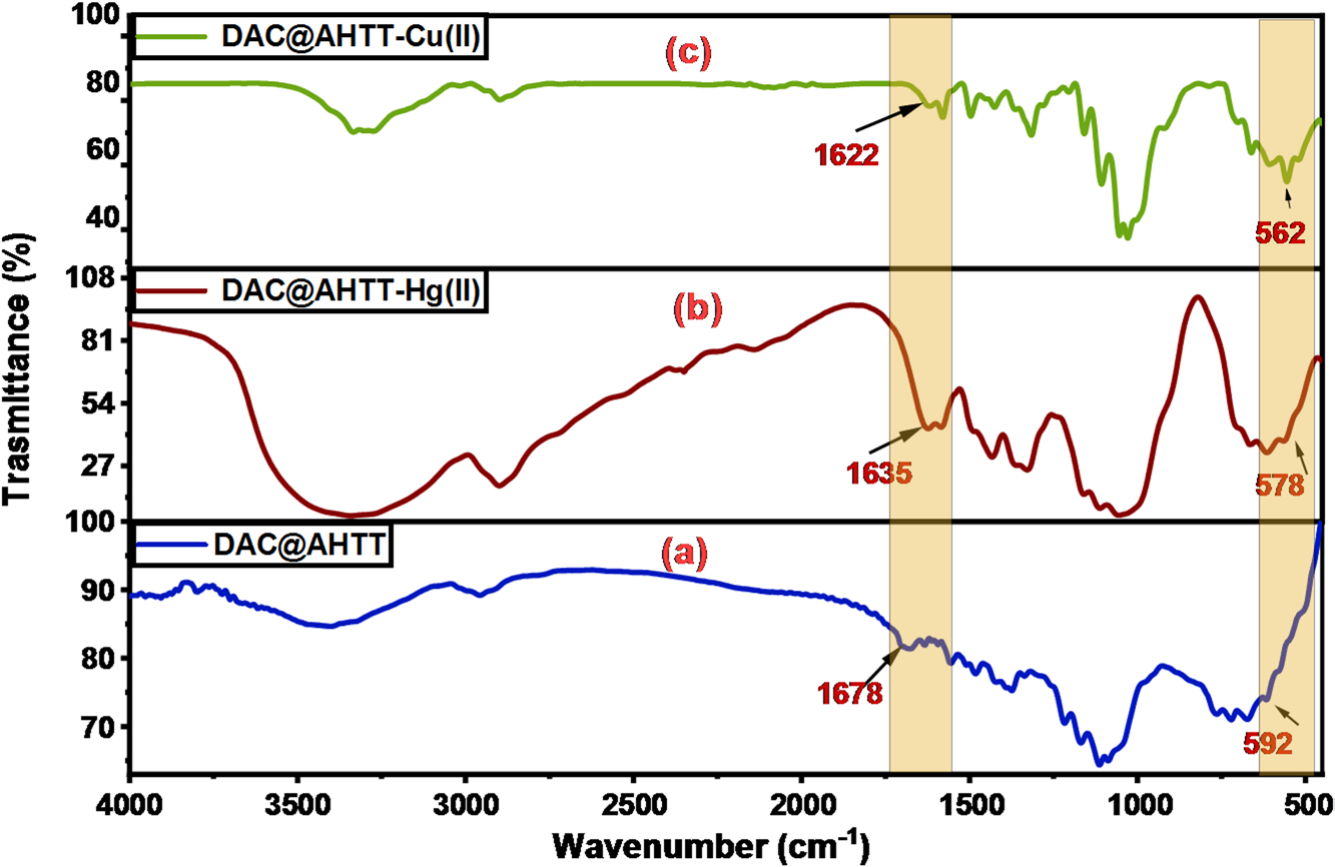
FTIR spectra of (**a**) DAC@AHTT adsorbent, (**b**) DAC@AHTT-Hg(II), and (**c**) DAC@AHTT-Cu(II).

**Fig. 19:**
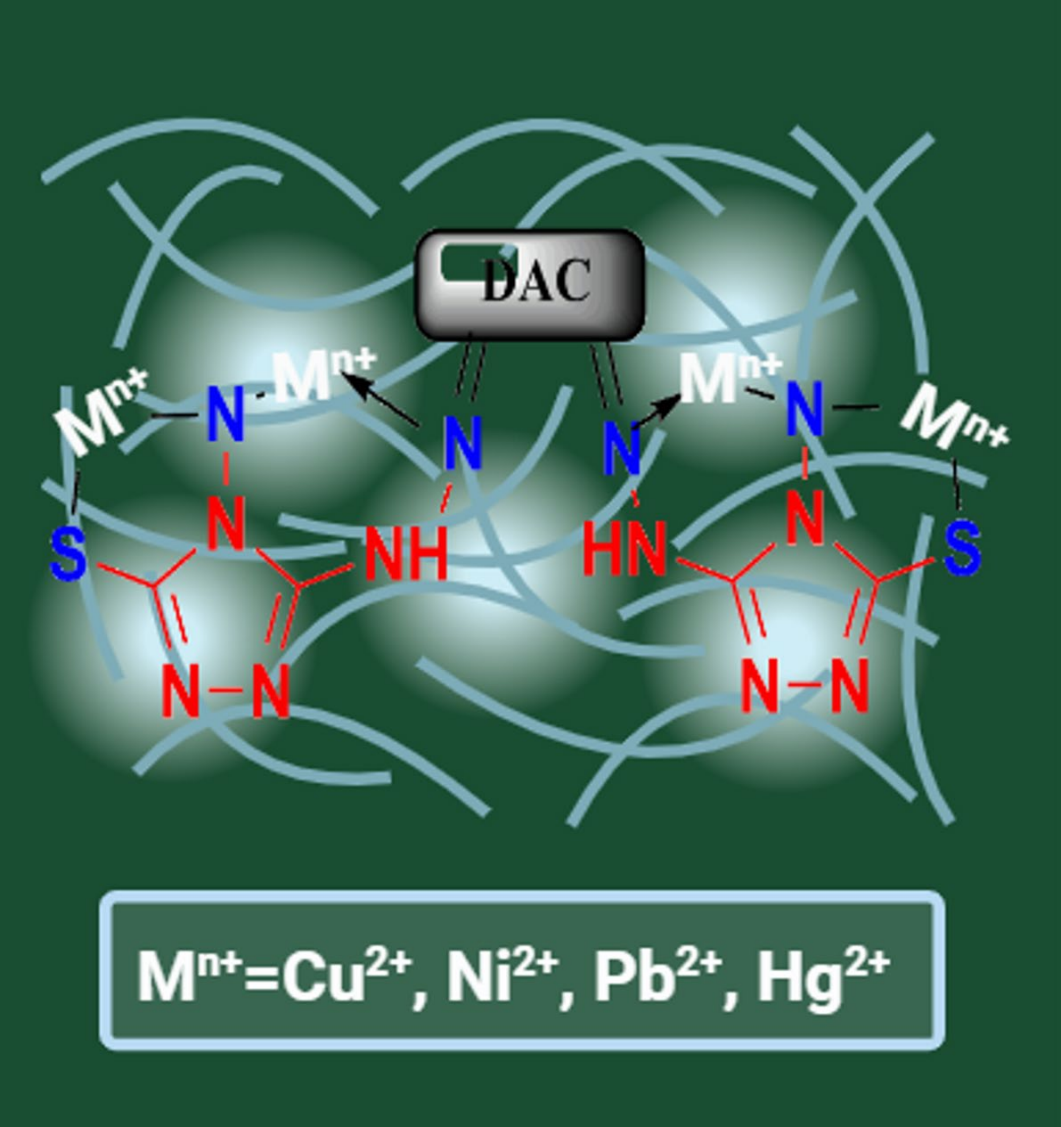
Plausible mechanism of sorption of heavy metals onto DAC@AHTT adsorbent.

To further ensure the DAC@AHTT adsorption behavior in the multi-heavy metal system, Cu(II) has been adsorbed individually under optimum conditions as part of the investigated multicomponent system. The DAC@AHTT-Cu(II) FT-IR spectrum (Fig. 18c) was obtained and compared with those of DAC@AHTT (Fig. 18a) and DAC@AHTT-Hg(II) (Fig. 18b) to evaluate the Cu(II) adsorption behavior with DAC@AHTT. It was noticed that the band of -C = N shifted after the Cu(II) adsorption from 1678 to 1622 cm^-1^. Moreover, the -NH bands also shifted. Furthermore, the -C-S group shifted from 592 to 562 cm^-1^, exhibiting higher intensity than that of the DAC@AHTT adsorbent^74^. Also, it was observed that the changes after the Cu(II) adsorption are the same as those of Hg(II). These evidences led us to assess the Cu(II) complexation behavior with the DAC@AHTT, Fig. 19, as follows:

1. Forming a Five-ring complex through the -SH and -NH_2_.
2. Forming a six-ring complex through the tertiary amine lone pair and the -NH_2_ group.

The DAC@AHTT’s BET surface area was found to be 42.4 m².g^-^¹, which is considered modest compared to other nanostructured adsorbents. The high Hg(II) uptake can be returned to the chemisorption through -S-H, -C = N, and -NH_2_ active groups. These active groups are known for their high affinity toward Hg(II) through coordination and complexation, resulting in the formation of strong Hg-S and Hg-N bonds^14^. Moreover, the adsorption efficiency of DAC@AHTT is primarily controlled by the chemical nature and accessibility of its active sites, rather than by its total surface area. Other published investigations have reported similar observations for other thiol- and amine-functionalized cellulose or polymeric adsorbents, where strong chemisorption interactions compensated for the relatively low specific surface area. Algeiri et al. reported a sulfhydrylated cellulose with an uptake of 1325 mg.g^-1^ for Hg(II) with a low surface area of 18 m^2^.g^-1^^109^. Moreover, a DAC@GuTSC cellulose-based adsorbent with a surface area of 3.038 m² g ¹ and Hg(II) uptake of 94 mg.g^-1^ has been reported by Akl et al.^53^.

To evaluate the stability of the DAC@AHTT, the DAC@AHTT FTIR spectra after three subsequent cycles of the adsorption-desorption are presented in Fig.S1. It was observed that the FTIR spectra do not show significant changes, especially the imine characteristic band at 1678 cm^-1^, after the three cycles. These observations confirm the stability of the C = N and the overall DAC@AHTT structure.

### Plackett–Burman design (PBD) experimental studies

 Table 12 illustrates the application of DAC@AHTT adsorbent for the adsorption of a multi-heavy metals polluted system containing a mixture of (Hg(II), Pb(II), Cu(II), and Ni(II)). The existing multicomponent adsorption process was conducted using different levels of the investigated heavy metals, with initial concentrations of 25 mg.L^− 1^ and 50 mg.L^− 1^ at -1 and + 1 levels, respectively^70^. It was demonstrated that the adsorption efficiency for the investigated heavy metals utilizing the prepared DAC@AHTT adsorbent was as follows: Cu(II) > Hg(II) > Pb(II) > Ni(II). The results in Table 12 demonstrate that Cu(II) achieved the highest removal efficiency among the other presented metal ions, with a capacity in the range of 24.80-35.27 mg.g^− 1^. Moreover, Hg(II) and Pb(II) achieved removal capacities in the ranges of 22.00-29.80 mg.g^− 1^ and 16.10–19.10 mg.g^− 1^, respectively. Ni(II) achieved the lowest removal capacity, ranging from 4.14 to 7.90 mg.g^− 1^. It was discovered that the mechanism of pollutants’ adsorption employing DAC@AHTT adsorbent in a multi-metal ion-contaminated system was entirely distinct from that in a single-metal ion-contaminated system. Moreover, the initial concentration of metal ions, ionic radius, polarizability, and inhibitory influence all play a crucial role in metal removal in multicomponent systems^110^. The ionic radius of Cu(II), 73 pm, is lower than that of Hg(II), 102 pm, which is lower than that of Pb(II), 119 pm. That may be the primary reason for the higher adsorption capacity of Cu(II) compared to Hg(II) and Pb(II). While Ni(II) has a lower ionic radius (69 pm) than Cu(II), Hg(II), and Pb(II), it has the lowest removal capacity among them^111^. This may be returned to the polarizability, as Ni(II) has the lowest value among the investigated metal ions. The more polarizable the ions, the stronger the interaction with soft donor atoms, such as S and N. As Ni(II) is considered a hard acid, and according to the theory of soft and hard acids and bases, the soft acids/soft bases interaction is more substantial than the hard acids/soft bases one^112^.

**Table 12:** Plackett–Burman experimental design matrix presenting different combination levels of the heavy metals in the evaluation of the removal of heavy metals (mg.g^− 1^) by DAC@AHTT.

Trials	Removal capacity (mg.g^− 1^)			
	Hg(II)	Pb(II)	Cu(II)	Ni(II)
1	23.08	16.80	29.54	4.80
2	27.50	17.95	25.00	5.10
3	22.90	17.10	27.90	4.14
4	24.20	16.45	29.65	5.08
5	26.50	17.20	25.00	4.90
6	23.10	16.10	29.70	4.30
7	22.30	17.14	32.08	6.80
8	22.00	17.35	35.27	7.64
9	24.50	18.45	24.95	7.54
10	29.80	17.80	24.80	5.20
11	24.20	18.60	24.91	7.70
12	24.80	19.10	25.00	7.90

### ANOVA statistical analysis

**Fig. 20:**
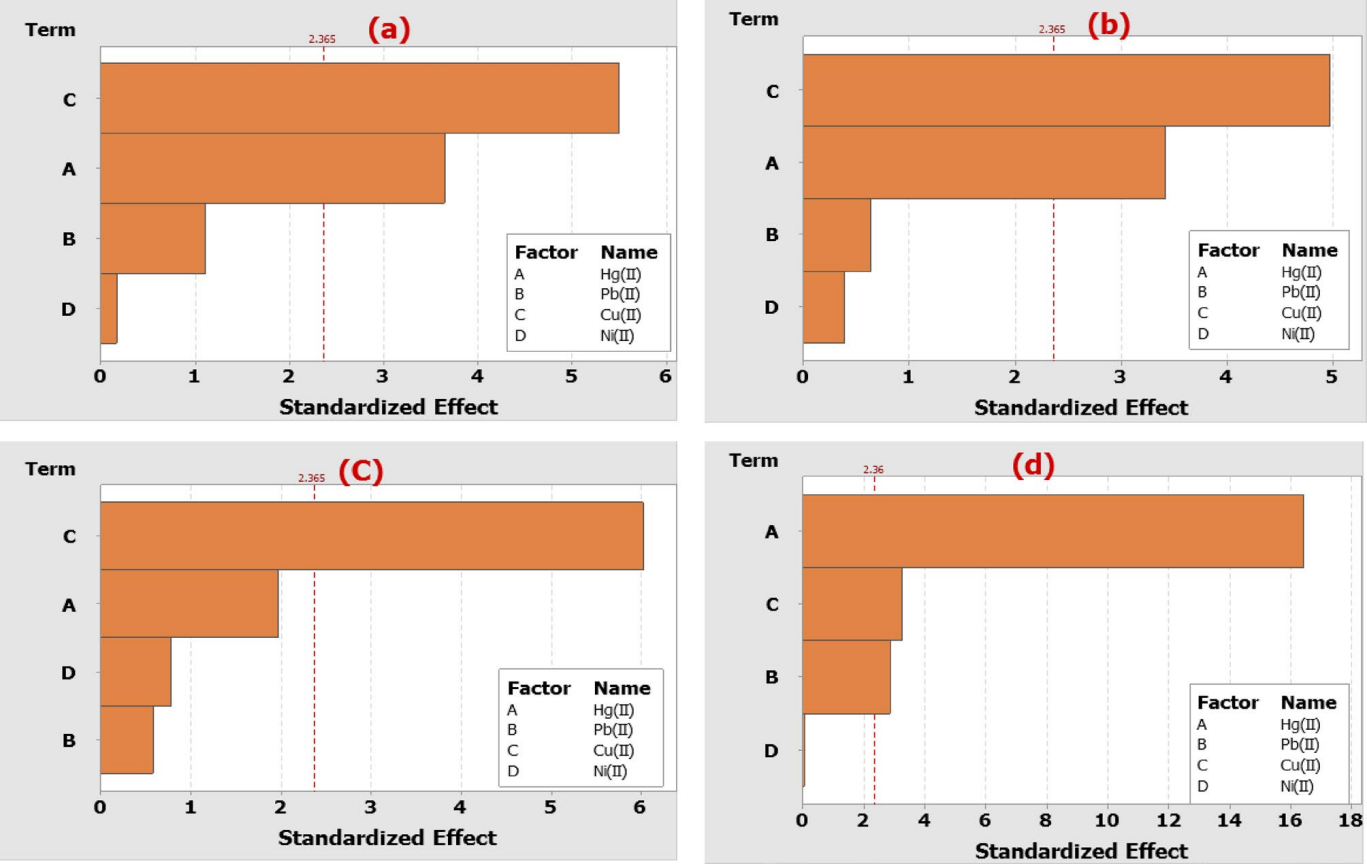
Pareto chart showing the effect of different heavy metals on each other’s removal by biosorbent: (**a**) effect of Hg(II), Pb(II), Cu(II), and Ni(II) on the adsorption of Hg(II), (**b**) effect of Hg(II), Pb(II), Cu(II), and Ni(II) on the adsorption of Pb(II), (**c**) effect of Hg(II), Pb(II), Cu(II), and Ni(II) on the adsorption of Cu(II), and (**d**) effect of Hg(II), Pb(II), Cu(II), and Ni(II) on the adsorption of Ni(II).

 Table 13 presents the statistical analysis of the investigated heavy metals (Hg(II), Pb(II), Cu(II), and Ni(II)) removal capacity data using the DAC@AHTT adsorbent, as determined by the ANOVA approach. The obtained Pareto charts, Fig. 20, indicate the individual metal effects on each investigated metal in the multicomponent system, represented by horizontal bars. The deviation in the final results can be well illustrated by the high R² and low P-values, which indicate the accuracy and efficiency of the multicomponent system, as well as a high degree of correlation between the experimental and predicted results. From the findings in Table 13, it can be concluded that Cu(II) negatively affects the adsorption of Hg(II), Pb(II), and Ni(II) using DAC@AHTT, with P-values of 0.001, 0.002, and 0.014, respectively. Therefore, Pb(II) has a significant inhibitory impact on the Ni(II) adsorption with a P-value of 0.024. Hg(II) has a highly significant negative influence on the Cu(II) adsorption (P-value of 0.00). The significant effect suggests a competitive manner. Hg(II) and Cu(II) have significant positive influences on their own removal, with P-values of 0.008 and 0.001, respectively, indicating their direct involvement in the removal capacity and response. The inhibitory influence of metal ions in the mixtures may be due to the screening action produced by the metals that exist in the solution^113,114^. The factors with P-values below 0.05 were considered statistically significant, confirming which metals exert synergistic or antagonistic effects on Hg(II) removal.

**Table 13:** Statistical analysis of Plackett-Burman design experiment for heavy metals removal by DAC@AHTT.

	Coefficients		T-value			*P*-value	
**For Hg(II)**							
Hg(II)	1.254		3.64			0.008	
Pb(II)	-0.375		-1.11			0.305	
Cu(II)	-1.862		-5.50			0.001	
Ni(II)	0.057		0.17			0.873	
**For Pb(II)**							
Hg(II)	-0.420		-3.42			0.011	
Pb(II)	-0.077		-0.64			0.544	
Cu(II)	-0.602		-4.97			0.002	
Ni(II)	-0.047		-0.38			0.714	
**For Cu(II)**							
Hg(II)	-0.985		-1.97			0.090	
Pb(II)	-0.286		-0.58			0.581	
Cu(II)	2.972		6.02			0.001	
Ni(II)	0.391		0.78			0.461	
**For Ni(II)**							
Hg(II)	-1.2830		-16.44			0.000	
Pb(II)	-0.2207		-2.87			0.024	
Cu(II)	-0.2507		-3.26			0.014	
Ni(II)	-0.0030		-0.04			0.970	

ANOVA							
	DF	Adj SS		Adj MS	F-value		*p* - value
**For Hg(II)**							
Regression	4	49.8824		12.4706	9.66		0.006
Residual	7	9.0395		1.2914			
Total	11	58.9219					
R^2^	0.8466						
**For Pb(II)**							
Regression	4	7.76537		1.94134	11.77		0.003
Residual	7	1.15410		0.16487			
Total	11	8.91947					
R^2^	0.8706						
**For Cu(II)**							
Regression	4	113.987		28.4967	10.39		0.005
Residual	7	19.194		2.7420			
Total	11	133.181					
R^2^	0.8559						
**For Ni(II)**							
Regression	4	22.9648		5.7412	86.44		0.000
Residual	7	0.4649		0.0664			
Total	11	23.4297					
R^2^	0.9802						

### Sustainability and environmental aspects of the DAC@AHTT adsorbent

Many published adsorbents have been studied for their capability to remove Hg(II). DAC@AHTT material reveals many advantages when compared to other published adsorbents. The first advantage is the sustainability, as DAC@AHTT is an environmentally friendly choice for the Hg(II) adsorption. It is a cellulose-based adsorbent that is functionalized with the AHTT Schiff base, resulting in the introduction of N/S functional groups and enhanced Hg(II) selectivity, thereby reducing the need for high adsorbent doses. DAC@AHTT is a recyclable adsorbent that exhibits good recyclability up to the third cycle. The second advantage is its cost-effectiveness, as it is a cellulose-based, inexpensive adsorbent. The third advantage is the high adsorption capacity, which is superior to that of alternative adsorbents, as presented in Table 11. The fourth advantage is the DAC@AHTT accessibility.

The recognition of limitations, alongside environmental trade-offs, is significant. During the modification step of AHTT loading on the DAC and regeneration (organic solvents, acids/bases), reagents that may offset particular sustainability concerns, the stability of the imine bond must be validated under various conditions. Reproducibility, cost, and waste disposal when scaling up are essential considerations.

Compared to the literature, DAC@AHTT is situated between inexpensive, raw adsorbents (which are less costly but also less selective) and high-performance synthesized materials (which provide higher performance and capacity but are very costly).

### Future recommendations

More attention should be provided to the removal of various pollutants in multicomponent systems, as it simulates the actual case investigation of real water treatment. Moreover, statistical analysis for multi-systems should be investigated to estimate the effect of each pollutant on the removal of the other. As the XPS and TEM analyses were not obtained in the current investigation due to equipment limitations, such characterizations would provide deeper insight into materials surface morphology and chemical states of Hg(II) on DAC@AHTT. Throwing light on the use of eco-friendly, practical, low-cost, and available adsorbents should be obtained. The actual application to real waste samples should be taken into consideration.

## Conclusion

This investigation provides the novel DAC@AHTT and demonstrates its high efficiency toward Hg(II) adsorption from aqueous solutions, integrating both experimental and statistical optimization techniques. It was fabricated through DAC chemical modification using the AHTT ligand and characterized by various techniques, including SEM, FT-IR, CHNS, and BET. The DAC@AHTT demonstrated effective adsorption behavior with a q_e_ of 495.75 mg.g^− 1^ at a pH range of 6–8, following Langmuir and PSO, indicating monolayer chemisorption. Thermodynamics reveals the spontaneous and exothermic nature of adsorption. The DAC@AHTT was efficiently applied for the remediation of the Hg(II) from real polluted water sources. The DAC@AHTT exhibited a good recycling performance of 85% after three adsorption-desorption cycles, indicating its high structural stability. A combination of experimental and spectroscopic techniques illustrates the link between the DAC@AHTT structure and its adsorption capacity. It shows that N and S atoms are the main adsorption sites. The Plackett–Burman experimental approach and the DAC@AHTT were successfully employed to investigate the adsorption of Hg(II), Ni(II), Pb(II), and Cu(II) in multicomponent systems. Despite these promising results, the experiments were conducted in a lab setting, which may not accurately represent real wastewater matrices. Future endeavors will focus on extensive application, optimization of regeneration, and validation using authentic environmental samples.

## Supplementary Information

Below is the link to the electronic supplementary material.

Supplementary Material 1

## Data Availability

Data is provided within the manuscript or supplementary information files.
