# Supplementary material for: Novel aminothiazole grafted cellulose composite for efficient removal of Hg(II) from wastewater in single and multicomponent systems
Source: Sci Rep. 2025 Dec 29;15:45673. doi: 10.1038/s41598-025-31905-2 (PMC12753772; doi:10.1038/s41598-025-31905-2)
Supplement: Supplementary file 1 — Supplementary Material 1 [file 41598_2025_31905_MOESM1_ESM.docx]

# **Novel aminothiazole grafted cellulose composite for efficient removal of Hg(II) from wastewater in single and multicomponent systems**

**Magda A Akl^1^* and Aya G Mostafa^1^**

**^1^**Department of Chemistry, Faculty of Science, Mansoura University, Mansoura 31556, Egypt

* To whom correspondence should be addressed: Prof Magda Akl.

email magdaakl@yahoo.com

**Figure S1:** FTIR spectra of **(a)** DAC@AHTT and **(b)** DAC@AHTT after 3 cycles of adsorption-desorption.

**Table S1:** Optimal parameters of ICP-OES for determining Hg(II), Pb(II), Ni(II), and Cu(II).

| **Parameter Name** | | **Parameter Value** |
| --- | --- | --- |
| RF generator power (kW) | | 1.2 |
| Frequency of RF generator (MHz) | | 40.68 |
| Plasma gas flow rate (L min^−1^) | | 12 |
| Auxiliary gas flow rate (L min^−1^) | | 0.75 |
| Nebulizer pressure (kPa) | | 160 |
| Wavelength (nm) | Hg(II) | 194.2 |
|  | Pb(II) | 220.35 |
|  | Ni(II) | 231.6 |
|  | Cu(II) | 324.75 |
| Observed height (mm) | | 9 |
| Pump rate (rpm) | | 15 |

**Table S2**: The Material Safety Data Sheets (MSDS)

| **Material** | **Hazard classification** | **Precautions** |
| --- | --- | --- |
| **Cellulose powder** | Nonhazardous | Standard Lab handling |
| **NaCl** |  |  |
| **HCl** | Corrosive can cause severe burns and Inhalation hazards | Use PPE, careful handling (slow addition), and neutralizing before spills. |
| **H_2_SO_4_** |  |  |
| **NaOH** |  |  |
| **AHTT** | Flammable and irritant | Use a PPE and work under ventilation |
| **Ethanol** | Highly flammable | Use PPE and use in fume hood |
| **Methanol** | Extremely toxic and flammable |  |
